# The global, regional, and national alcohol-related colorectal cancer burden and forecasted trends: results from the global burden of disease study 2021

**DOI:** 10.3389/fnut.2024.1520852

**Published:** 2024-12-24

**Authors:** Jinfeng Yao, Guo Chen

**Affiliations:** Department of Oncology, Shuguang Anhui Hospital Affiliated to Shanghai University of Traditional Chinese Medicine (The First Affiliated Hospital West District of Anhui University of Chinese Medicine), Hefei, China

**Keywords:** colorectal cancer, alcohol, GBD, 2021, BAPC model, APC model

## Abstract

**Background:**

The mortality of colorectal cancer (CRC) is increasing year by year and poses a significant global health burden. Many studies have demonstrated that alcohol consumption is an important risk factor for CRC and is closely associated with malignant metastasis in CRC patients, which in turn leads to a poor prognosis.

**Methods:**

This study aimed to quantify the global, regional, and national burden of alcohol-related CRC between 1990 and 2021. First, numbers and age-standardized rates of deaths and disability-adjusted life years (DALYs) for alcohol-related CRC in 2021 were analyzed at different levels. Temporal trends in the burden of disease from 1990 to 2021 were analyzed through linear regression models. Finally, both Age-Period-Cohort (APC) models and Bayesian Age-Period-Cohort (BAPC) models were utilized to project the future burden of the disease for 2022–2046.

**Results:**

The global burden of disease for alcohol-related CRC is higher in 2021 compared to 1990. Male and older age groups are at high risk. Disease burden varies very much between Sociodemographic Index (SDI) regions, Global Burden of Disease (GBD) regions and countries. From 1990 to 2021, the number of cases increased, but the Age-Standardized Rate (ASR) decreased. The trends in disease burden predicted by the two models for 2022–2046 were not consistent.

**Conclusion:**

This study describes the burden of disease in alcohol-related CRC and emphasizes that alcohol is a non-negligible risk factor for CRC. In order to mitigate harm, we need to strengthen disease surveillance, early prevention, timely detection, and improved treatment measures, with different approaches and responses for different regions.

## 1 Introduction

In recent years, due to advances in medical diagnostic technology and widespread cancer screening, the diagnosis rate of colorectal cancer (CRC) has increased significantly (1). According to the World Health Organization cancer statistics, by 2022, CRC will have the third highest incidence rate of all cancers globally, with 1,926,425 new cases (age-standardized incidence rate of 10.7/100,000); and the second highest mortality rate of all cancers, with 904,019 new deaths related to CRC (age-standardized mortality rate of 4.7/100,000).^1^ The incidence of CRC worldwide is projected to increase to 2.5 million new cases by 2035 (2). The treatment prognosis of early-stage CRC patients is much better than that of middle-stage and late-stage patients (3). However, because it takes several years for colorectal polyps to develop into colorectal cancer, its early diagnosis rate is low (4). Therefore, early detection, screening and treatment can effectively reduce the burden of CRC and lower the mortality rate of CRC.

Risk factors for colorectal cancer include two main categories: genetic and environmental factors. With regard to genetic factors, approximately 10–20% of colorectal cancer patients have a positive family history and the degree of risk is influenced by the number and extent of related relatives and the age at which CRC is diagnosed (5, 6). Approximately 5–7% of CRC patients are also affected by well-defined hereditary colorectal cancer syndromes (including Lynch syndrome, familial colorectal cancer and polyposis syndromes) (7). As for lifestyle effects, smoking, alcohol intake, red meat intake, reduced vegetable intake and obesity are positively associated with CRC burden. On the contrary, increased physical activity, vitamin and dietary fiber intake were negatively associated with the development of CRC. In addition, drug trials, gender, type 2 diabetes and inflammatory bowel disease were also strongly associated with the development of CRC (8).

Alcohol consumption has been shown to be an important risk factor for colorectal cancer (CRC), and it is strongly associated with metastasis in patients with CRC, which is the main reason for the poor prognosis of patients (9, 10). There are many reports on the mechanisms by which alcohol causes CRC, such as polymorphisms affecting ethanol metabolizing enzymes, folate metabolizing pathways and gene repair (11, 12). It has also been reported that alcohol intake may significantly promote CRC metastasis through ethanol-mediated TGF-*β*/Smad/Snail axis (13). Authoritative reports show that alcohol intake in Chinese men is positively correlated with the risk of 28 diseases, including CRC. For every 280 g increase in average weekly alcohol intake, there is a 19% increase in colorectal cancer risk compared to non-drinkers (14).

Previous Global Burden of Disease (GBD) studies have investigated the colorectal burden profile, elucidating substantial differences in morbidity and mortality across geographic regions and age. The burden of CRC has also been analyzed dynamically from 1990 to 2019. Risk factors associated with CRC were also briefly described (15). However, these analyses mainly focused on colorectal cancer incidence alone and did not explicitly investigate the impact of alcohol factors on changes in colorectal cancer burden. Therefore, there is a need to assess the combined burden of colorectal cancer caused by alcohol intake and to update epidemiologic data with the most recent data, as well as to predict future trends.

This study will assess the global, regional, and national burden of alcohol-related colorectal cancer by analyzing mortality and disability-adjusted life years (DALYs) from 1990 to 2021. With the aim of providing important insights into the development of alcohol-related colorectal cancer epidemiology, it will inform colorectal cancer prevention strategies to reduce the growing burden of this disease.

## 2 Methods

### 2.1 Data sources

The Global Burden of Disease (GBD) 2021 study is the primary data source, which collects data from censuses, household surveys, civil registration and vital statistics, disease registries, health service use, satellite imaging, disease notifications, and other sources using Bayesian meta-regression modeling tools to model and estimate burdens under several scenarios for each country and region from 1990 to 2021 (16). GBD provides comprehensive estimates of death- and disability-adjusted life years by cause, age, sex, and location (17). The GBD study used a comparative risk assessment methodology to estimate the attributable burden of alcohol-related colorectal cancer using data from multiple sources (18, 19).

### 2.2 Statistical analysis

First, we report the number of alcohol-related colorectal cancer deaths and Disability-Adjusted Life Years (DALYs), and corresponding age-standardized rates (ASRs) for 1990 and 2021, respectively. The analysis was conducted globally and stratified by subtypes, including gender, age group, Socio-demographic Index (SDI) region [constructed based on three indicators ranging from 0 to 1, with larger SDIs indicating more developed (20)], GBD region, and country. We then analyzed global and different subtype trends from 1990 to 2021 to investigate the dynamics of the disease burden. By calculating the Estimated Annual Percentage Change (EAPC) values, we performed a hierarchical cluster analysis of the results, dividing the 54 GBD regions into four groups, including significant increase, significant decrease, remained stable or minor decrease, and minor increase. Finally, we utilized the Age-Period-Cohort (APC) model and Bayes age-period-cohort (BAPC) model to predict the burden of alcohol-related colorectal cancer from 2022 to 2046.

In this study, all statistical analyses and visualizations were performed using the R statistical software program (V.4.0.2). *p* < 0.05 was considered a statistically significant difference.

## 3 Results

### 3.1 The disease burden of alcohol-related colorectal cancer in 2021

An estimated 56,102 (95% UI 43738–69,936 million) deaths from alcohol-related colorectal cancer worldwide in 2021, with an age-standardized death rate (ASDR) of 0.66 cases per 100,000 (95% UI: 0.51–0.82) (Supplementary Table S3). In addition, the number of cases of DALYs was 1,425,324 (95% UI: 1122455–1,769,389), with an age-standardized DALYs rate (ASDAR) of 16.44 cases per 100,000 (95% UI: 12.95–20.43) (Supplementary Table S4).

From a gender perspective, the burden on male is more than four times that of female, both in terms of the number of death cases and the number of DALYs cases (Supplementary Figure S1; Supplementary Tables S3, S4). The number of deaths in male was 46,090 (95% UI: 35716–57,352) and the number of DALYs cases was 1,197,848 (95% UI: 932790–1,488,851); the number of deaths in female was 10,012 (95% UI: 7409–12,863) and the number of DALYs cases was 227,476 (95% UI: 174133–288,659) (Supplementary Figure S1; Supplementary Tables S3, S4).

An age-stratified analysis of death and DALYs for alcohol-related colorectal cancer from an age perspective is shown in Supplementary Figure S2. In 2021, age-standardized rates for deaths and DALYs generally increased with age. However, the number of cases of deaths and DALYs increases and then decreases with age. The number of deaths from alcohol-related CRC peaked at age 70–74 at 8192 (95% UI: 6520–10,243); the number of DALYs from alcohol-related colorectal cancer peaked at age 65–69 at 199088 (95% UI: 158106–247,424) (Supplementary Figure S2; Supplementary Tables S3, S4).

When analyzed from an SDI perspective, high SDI had the highest ASR and number of cases, with the following values: an ASR of 1.08 (95% UI: 0.85–1.32) for deaths, an ASR of 27.17 (95% UI: 21.81–32.97) for DALYs, a number of cases of 22,924 (95% UI: 17,836-2,098) for deaths, and a number of cases of 513,549 for DALYs (95% UI: 40,8,432-624,443). However, the minimum values of ASR and number of cases were localized to different SDI. Low-meddle SDI has the smallest ASR and low SDI has the least number of cases (Supplementary Figure S3; Supplementary Tables S3, S4).

In the 54 GBD regions, the number of death cases and DALYs due to alcohol-related colorectal cancer deaths in Advanced Health System were the highest at 31,678 (95% UI: 24,529-38,912) and 720,062 (95% UI: 565,673-876,030), respectively; whereas Oceania was the lowest number at 5 (95% UI: 3–7) and 171 (95% UI: 110–234). In addition, the ASR of deaths from alcohol-related CRC and ASR of DALYs were the highest in Central Europe with 1.86 (95% UI: 1.43–2.32) and 46.57 (95% UI: 36.49–57.33), respectively, while Northern Africa was the lowest with 0.02 (95% UI: 0.01–0.02) and 0.52 (95% UI: 0.36–0.73) (Supplementary Figure S4; Supplementary Tables S3, S4).

**Figure 1:**
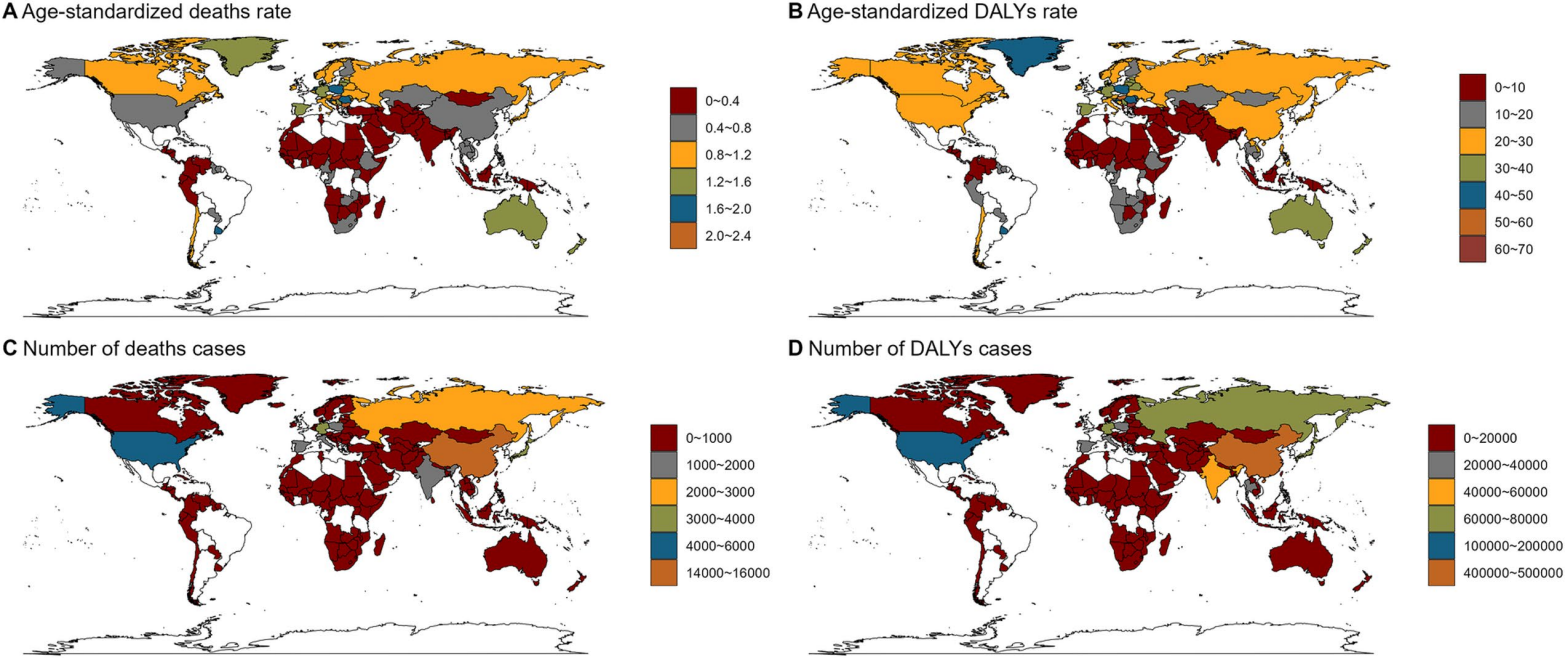
Numbers and age-standardized rates of alcohol-related colorectal cancer-related deaths and DALYs across countries and territories in 2021. **(A)** Age-standardized deaths rates of alcohol-related colorectal cancer across countries and territories in 2021; **(B)** Age-standardized DALYs rates of alcohol-related colorectal cancer across countries and territories in 2021; **(C)** Numbers of deaths cases of alcohol-related colorectal cancer across countries and territories in 2021; **(D)** Numbers of DALYs cases of alcohol-related colorectal cancer across countries and territories in 2021. DALYs, disability-adjusted life years.

The disease burden of alcohol-related colorectal was analyzed for each country. In 2021, China had the highest number of deaths cases of alcohol-related colorectal cancer, while American Samoa, Bhutan, Brunei Darussalam, Cook Islands, Djibouti, Kiribati, Maldives, Marshall Islands, Mauritania, Micronesia, Nauru, Niue, Northern Mariana Islands, Palau, Samoa, Solomon Islands, State of Kuwait, Sudan, Sultanate of Oman, Tokelau, TongaTuvalu, Union of the Comoros and Vanuatu have little to no burden of the disease. Regarding ASR, Bulgaria has the highest (Figure 1; Supplementary Tables S3, S4).

### 3.2 Temporal trend for alcohol-related colorectal cancer burden from 1990 to 2021

**Figure 2:**
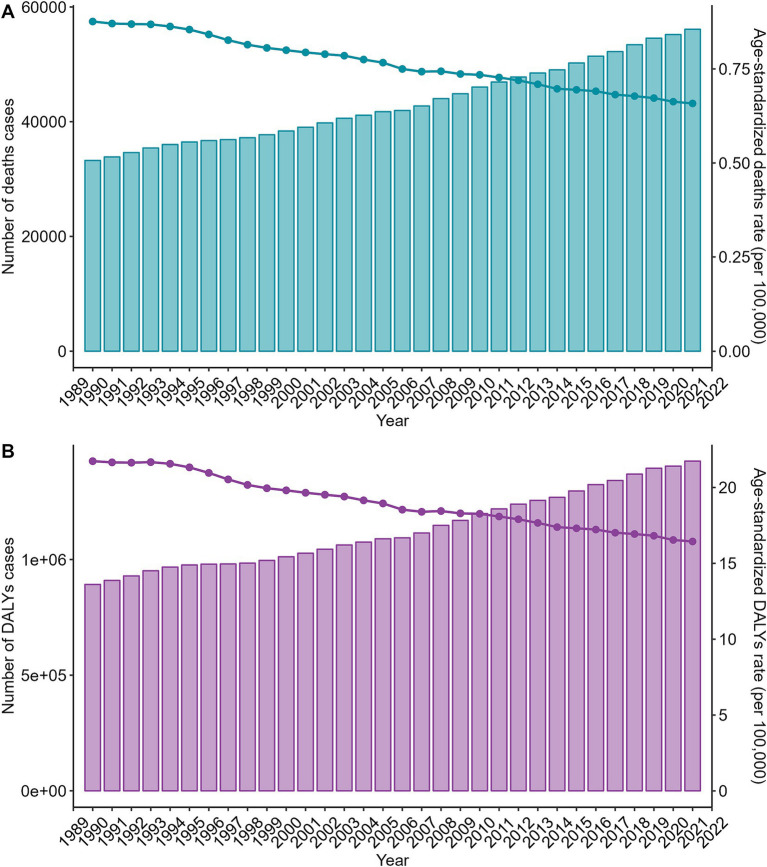
Trends in the numbers and age-standardized rates of alcohol-related colorectal cancer-related deaths and DALYs globally from 1990 to 2021. **(A)** Trends in the number of deaths cases and age-standardized deaths rate of alcohol-related colorectal cancer-related deaths globally from 1990 to 2021; **(B)** Trends in the number of DALYs cases and age-standardized DALYs rate of alcohol-related colorectal cancer-related deaths globally from 1990 to 2021. DALYs, disability-adjusted life years.

To analyze trends in the burden of disease for alcohol-related colorectal cancer from 1990 to 2021. The disease shows an upward trend in both deaths and DALYs. Globally, the number of deaths doubled from 33,239 (25,537-4,130) in 1990 to 56,102 (43,738-6,9,936) in 2021. The number of cases of DALYs increased from 892,190 (707,015-10,890,013) to 142,532,324 (11,224,555-17,693,989). However, ASDR and ASDAR showed a decreasing trend. ASDR decreased from 0.88 (0.66–1.09) in 1990 to 0.66 (0.51–0.82), and ASDAR decreased from 21.74 (16.95–26.65) in 1990 to 16.44 (12.95–20.43) in 2021 (Figure 2; Supplementary Tables S3, S4).

Looking at trends in the burden of disease over time from a gender perspective, the change in ASR for both sexes was consistent with the overall population, showing a downward trend. The number of deaths and DALYs for males is also consistent with the overall population, although the number of deaths and DALYs for females shows an increasing trend from 1990 to 2021, with only very slight changes (Supplementary Figure S5; Supplementary Tables S3, S4). The trends in burden of disease indicators for all age groups were nearly identical to the global except for the 95+ age group, which showed an increasing trend in ASR from 1990 to 2021, which was inconsistent with the overall ASR trend, as shown in Supplementary Figure S6 and Supplementary Tables S3, S4. The number of deaths and the number of cases of DALYs in each SDI were consistent with the overall trend, and the High SDI and High-middle SDI ASR trends were also consistent with the overall, but the ASR trends for the remaining SDIs were in the opposite direction from the overall (Supplementary Figure S7; Supplementary Tables S3, S4).

**Figure 3:**
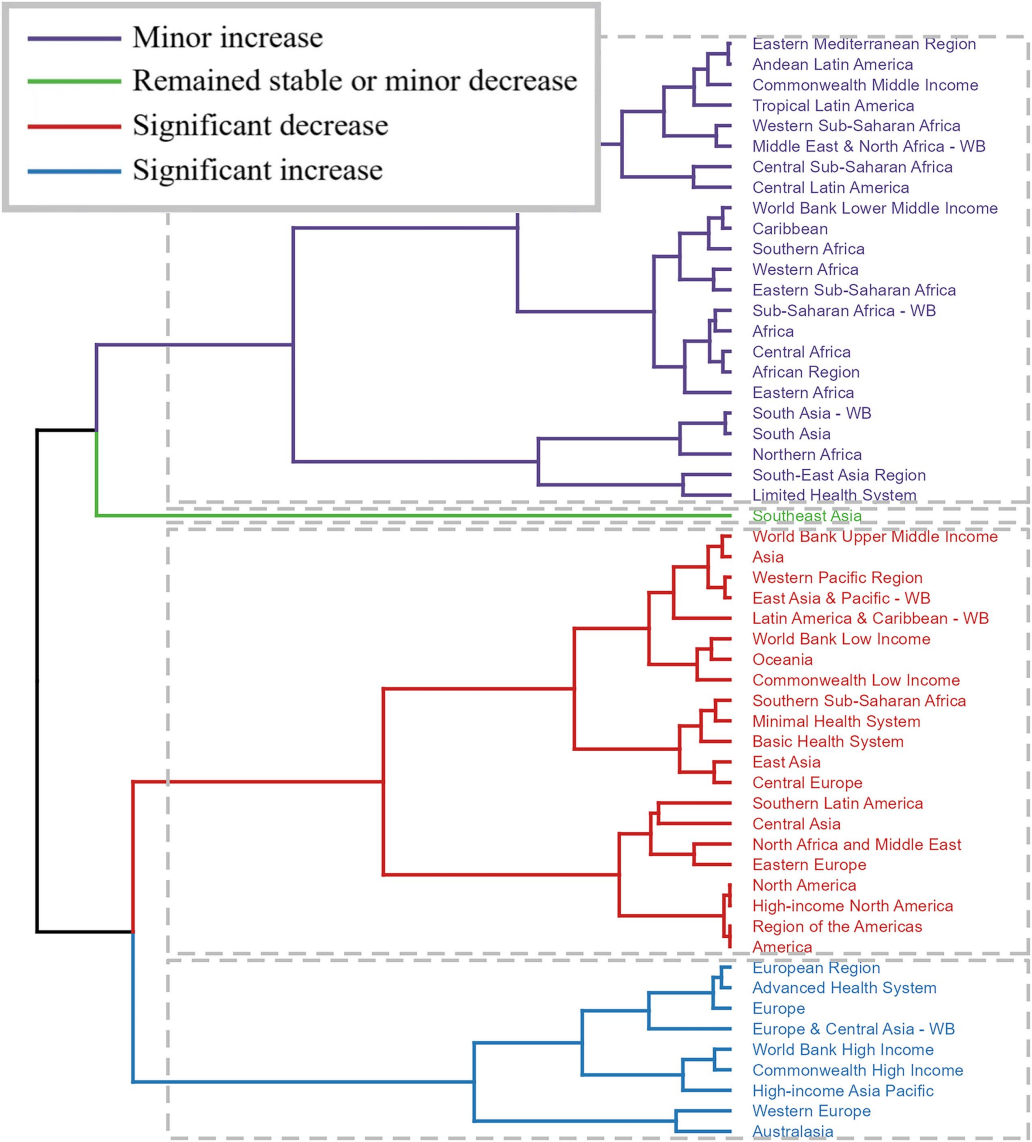
Results of cluster analysis based on the EAPC values of alcohol-related colorectal cancer-related age-standardized rates for deaths and DALYs from 1990 to 2021. EAPC, estimated annual percentage change; DALYs, disability-adjusted-life-years.

In order to better visualize the change in the burden of alcohol-related colorectal cancer from 1990 to 2021 in the 56 GBD regions, a stratified cluster analysis was conducted. As shown in Figure 3, ASR showed a significant increase in nine countries including European Region, Advanced Health System, Europe, Europe & Central Asia-WB, World Bank High Income, Commonwealth High Income, High-income Asia Pacific, Western Europe and Australasia, and a significant decreasing trend in another 21 countries including: World Bank High Income, Asia, Western Pacific Region, East Asia & Pacific-WB, Latin America and Caribbean-WB, World Bank Low Income, Oceania, Commonwealth Low Income, Southern Sub-Saharan Africa, Minimal Health System, Basic Health System, East Asia, Central Europe, Southern Latin America, Central Asia, North Africa and Middle East, Eastern Europe, North America, High-income North America, Region of the Americas and America.

**Figure 4:**
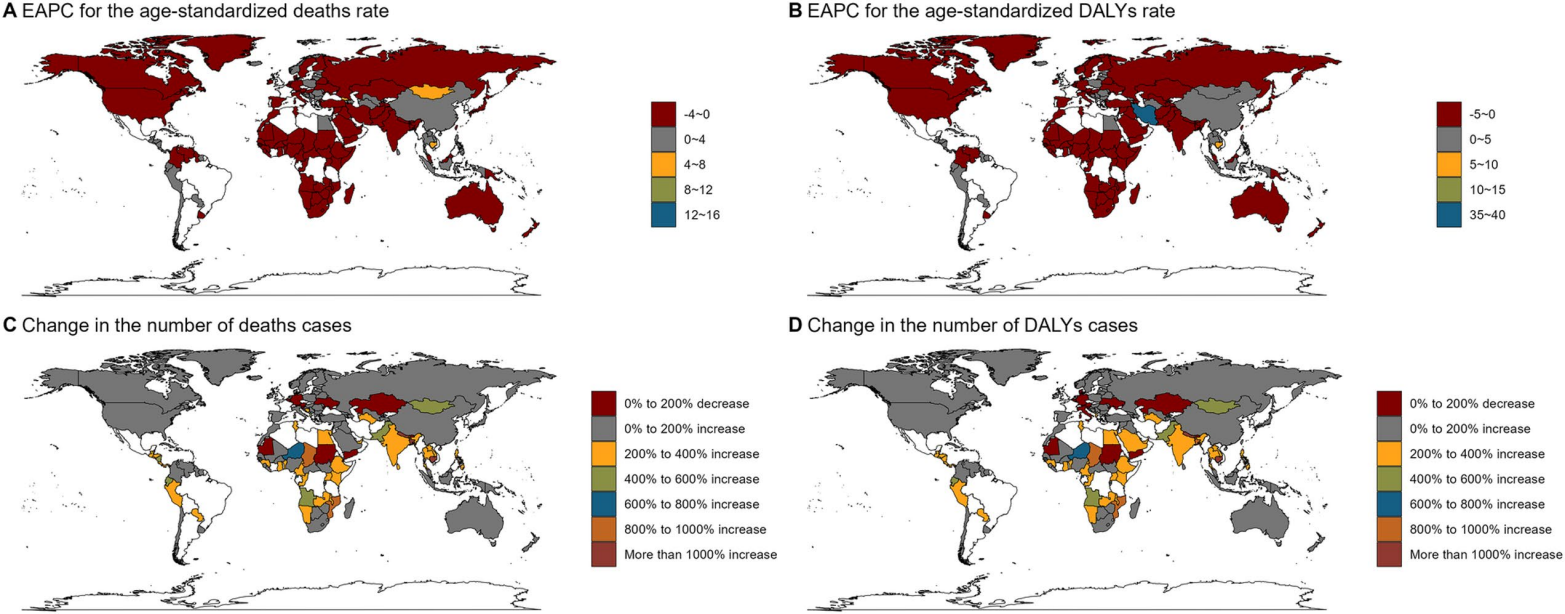
The EAPC of alcohol-related colorectal cancer-related ASR and the relative change in the numbers of deaths and DALYs between 1990 and 2021. **(A)** The EAPC of age-standardized deaths rate for alcohol-related colorectal cancer between 1990 and 2021; **(B)** The EAPC of age-standardized DALYs rate for alcohol-related colorectal cancer between 1990 and 2021; **(C)** The change in the number of alcohol-related colorectal cancer deaths between 1990 and 2021; **(D)** The change in the number of alcohol-related colorectal cancer DALYs between 1990 and 2021. EAPC, estimated annual percentage change; ASR, age-standardized rate; DALYs, disability-adjusted-life-year.

Trends in burden of disease varied across countries. Iran had the highest increase in ASR (EAPC = 39.88, 95% CI: 32.44–47.73) and Bahrain had the highest decrease in ASR (EAPC = -3.12, 95% CI: −3.31-2.93). Similarly, Iran had the highest growth rate in ASDR (EAPC = 37.4, 95% CI: 30.56–44.59) and Bahrain had the most significant decline in ASDR (EAPC = -3.01, 95% CI: −3.21-2.81) (Figure 4; Supplementary Tables S3, S4).

### 3.3 The predicted results of disease burden for alcohol-related colorectal cancer from 2022 to 2050

According to the results predicted by the APC model, the number of deaths and the number of DALYs in the future showed a significant upward trend in males, a slow upward trend in females, and an overall slight downward trend in ASR (Supplementary Figure S8). However, in the results predicted by the BAPC model, the number of deaths and the number of cases of DALYs in both males and females showed an almost constant trend, while the ASR showed a decreasing trend, and the slope of the change curve in males was steeper than that in females (Supplementary Figure S9).

## 4 Discussion

This study provides the first systematic and comprehensive description of the global alcohol-related CRC disease burden based on the GBD 2021 dataset, and gives the global burden of disease and its share for different SDI levels, geographic regions, sexes, and age groups over a 30-year period from 1990 to 2019, with projections for 2022 to 2046.

The burden of disease due to alcohol-induced CRC is significantly higher in 2021 compared to 1990 and has become a major hazard threatening human life and health. Our results show that the number of deaths and DALYs from alcohol-related CRC is steadily increasing globally from 1990 to 2021, and consistent with previous research, factors including obesity, physical inactivity, poor diet, alcohol consumption, tobacco use, and surgical site infections (SSI) all significantly contribute to the increasing mortality from CRC (21, 22). Analyzed from the ASR point of view, the ASDR and ASDAR of the disease showed a decreasing trend. This is consistent with the findings of a previous study which postulated that early detection of colorectal cancer through screening, cancer registries, and technological improvements to normalize early referral is a significant contributor to the decline in ASR (23).

Differences in physiological make-up and lifestyle habits due to gender are also an important factor influencing the distribution of disease mortality. Our results suggest that the number of deaths and DALYs from alcohol-related CRC is approximately 4–5 times higher in male than in female, while the trend of ASR changes in both men and women is consistent with the overall population. The first international report on colorectal cancer published in the 1960s showed a higher age-standardized incidence in female than in male, but since then there has been a much higher upward trend in male than in female, giving rise to the present epidemiological profile (24). The significantly higher CRC disease burden in male may be due to the high prevalence of visceral adiposity and the relatively higher prevalence of smoking. It has also been shown that alcohol consumption and smoking have a greater impact on DALYs of CRC in male than in female (18, 25, 26). In addition to risk factors, endogenous estrogen secretion in female has been reported to be protective against CRC development, and oral contraceptives have the potential to reduce CRC risk (27, 28). All these results suggest that gender regulates the biological clock significantly differently in males and females, and in-depth studies are needed to explore the mechanisms behind gender differences that affect CRC morbidity and mortality, and to provide a gender-differentiated approach to disease prevention, diagnosis, and treatment.

Similar to previous results, our age-stratified analysis of deaths and DALYs from alcohol-related CRC in 2021 showed that the burden of disease increased significantly with age (29–31), with the highest number of deaths and DALYs clustered in the 65–74 year age group. This positive association of disease burden with age implies a cumulative risk effect of alcohol exposure. These results illustrate the value of timely control of risk factors in effectively reducing the overall CRC disease burden, especially in older age groups.

This study also analyzed the burden of disease of alcohol-related CRC in different SDI regions. The High SDI region was at the top of the list in terms of both cases and burden of disease, and the trends in the number of cases and ASR in the High SDI region were consistent with those of the overall during the period of 1990–2021. However, due to early detection, intervention, and treatment, ASRs in these areas are decreasing yearly and referral normalization is increasing. Similar to previous results (32), in contrast to the overall trend, the age-standardized deaths rates (ASRs) of the disease in middle-SDI, lower-middle SDI, and low-SDI regions show a slow upward trend. It is hypothesized that these areas are experiencing the pre-variation phase of disease burden in High SDI areas due to horizontal developmental constraints. All these results show the complex relationship between socioeconomics and disease burden.

The findings also analyzed the disease burden of alcohol-related CRC at the level of geographic differences. Advanced Health System regions led the way in both deaths and DALYs, followed by World Bank High Income regions, World Bank Upper Middle Income regions, and Asia, with China being highly visible in the map distribution of deaths and DALYs. And with regard to ASR, Center Europe is located at the highest point. Hierarchical cluster analysis also categorized ASRs according to whether they were increasing or decreasing, significant or non-significant, tagging each region and emphasizing the need for individualized development of interventions. Specifically, Europe, High-Income Asia Pacific, Australasia, and Commonwealth High Income showed an increasing trend in alcohol-related CRC, whereas East Asia, Central Europe, and North America, among others, showed a significant decreasing trend in CRC. At the country level, Iran showed the most significant increase in disease burden, while Bahrain showed the greatest decrease. This is consistent with previous findings that due to rapid economic growth and industrialization, developing countries are introducing a westernized lifestyle characterized by unhealthy diets (less fruits and vegetables, more red and processed meats), less physical activity, and the development of bad habits (alcohol and smoking) (23, 33). These changes in lifestyle habits cause an increase in the mortality of several diseases, including colorectal cancer.

In order to develop more effective treatment programs for alcohol-related colorectal cancer, we took two models, APC and BAPC, and projected the burden of disease from APC and BAPC for the years 2022–2046. According to the APC prediction, the number of cases of alcohol-related CRC will increase every year, but the ASR is smoother. Due to the aging population and increasing life expectancy, improved screening and testing, and lifestyle changes, some studies have predicted a further increase in colorectal cancer incidence (23). Interestingly, the BAPC results showed that the number of cases in the next 30 years showed horizontal fluctuations and may even decrease, while the ASR showed a decreasing trend. The inconsistency between the results of the two models reflects the confounding nature of factors in the disease burden of alcoholic CRC, which cannot well predict the future direction. Our study focuses on the deaths of alcohol-induced colorectal cancer, and there have been many studies showing that alcohol consumption is one of the main factors in the increase of DALYs in CRC (34–36). Therefore, increasing the popularization of the dangers of alcohol consumption with limiting the use of alcohol is an effective measure to reduce the disease burden of CRC.

The limitations of this study are similar to other GBD studies (16). First, because the raw data were obtained from civil registration, vital statistics, and hospital records, the completeness of information from these systems affected the accuracy of the estimates. For example, the scarcity of data in areas with poorly developed health care level systems does not reflect well the true picture of their disease burden. Second, the GBD study covers a wide time frame, with changes in diagnostic and therapeutic approaches to CRC in most countries, as well as in a number of social and economic factors. As a result, disease mortality is more likely to be underestimated in earlier periods than in recent years, and this factor should be taken into account when interpreting changes in trends (23). Third, our projections consider alcohol alone, while many other risk factors that may contribute significantly to the burden of CRC are excluded, and this oversimplified model may undermine the accuracy and robustness of our projections.

## 5 Conclusion

From 1990 to 2021, the absolute number of alcohol-related CRCs has increased dramatically, posing a major threat to people’s health and well-being and placing an enormous burden on global health care systems. Our research is critical to achieving key targets of the Sustainable Development Goal on reducing the burden of cancer and other non-communicable diseases. To mitigate harm, we need to strengthen disease surveillance, early prevention, timely detection, improved treatment strategies and regionally specific measures.

## Data Availability

The original contributions presented in the study are included in the article/Supplementary material, further inquiries can be directed to the corresponding author.

## Footnotes

^1^ https://gco.iarc.fr/today/en
