# Supplementary figures and images for: The global, regional, and national alcohol-related colorectal cancer burden and forecasted trends: results from the global burden of disease study 2021
Source: Front Nutr. 2024 Dec 24;11:1520852. doi: 10.3389/fnut.2024.1520852 (PMC11704491; doi:10.3389/fnut.2024.1520852)

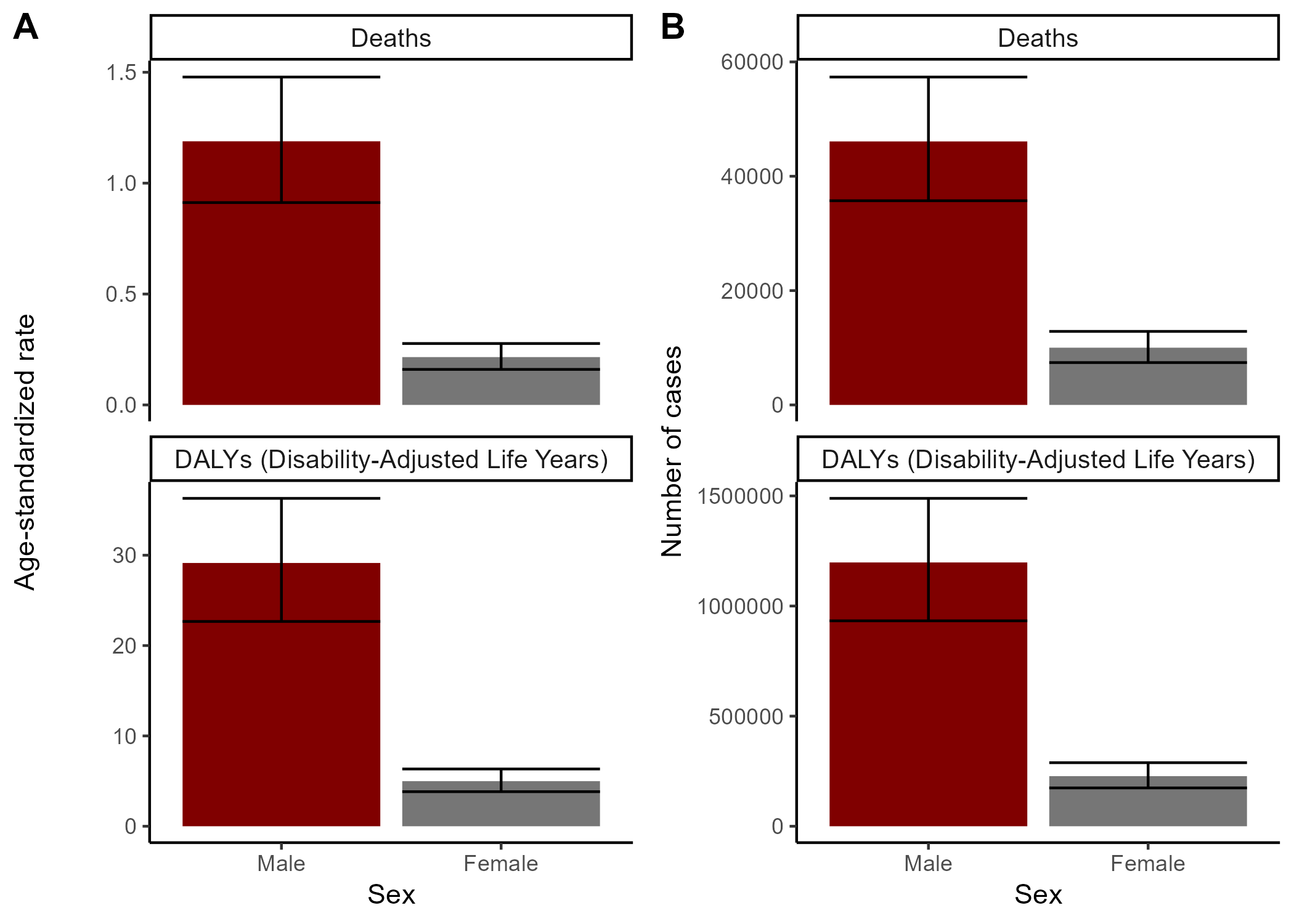

Supplement: Supplementary file 1 [file Image_1.tif]

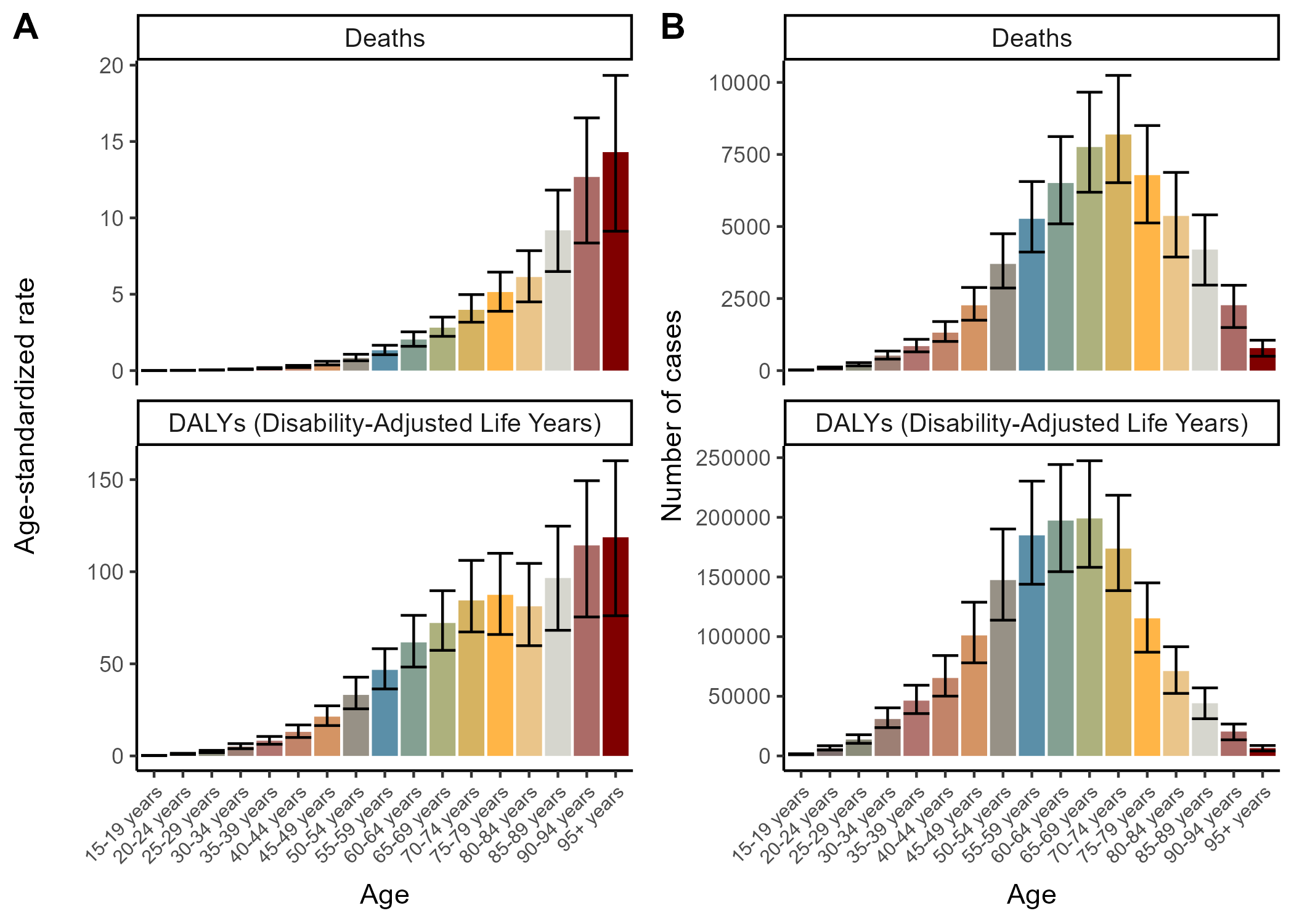

Supplement: Supplementary file 2 [file Image_2.TIF]

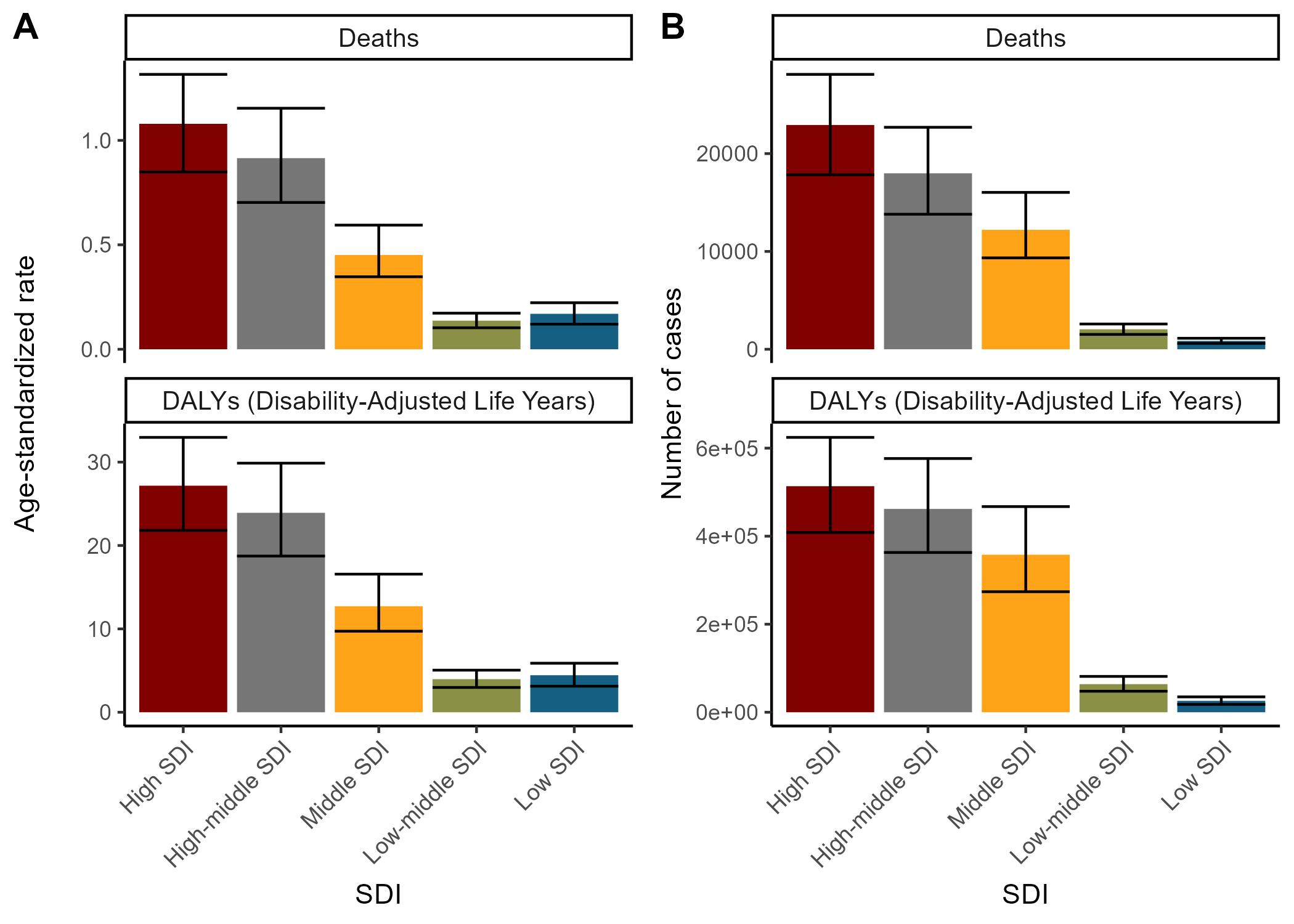

Supplement: Supplementary file 3 [file Image_3.TIF]

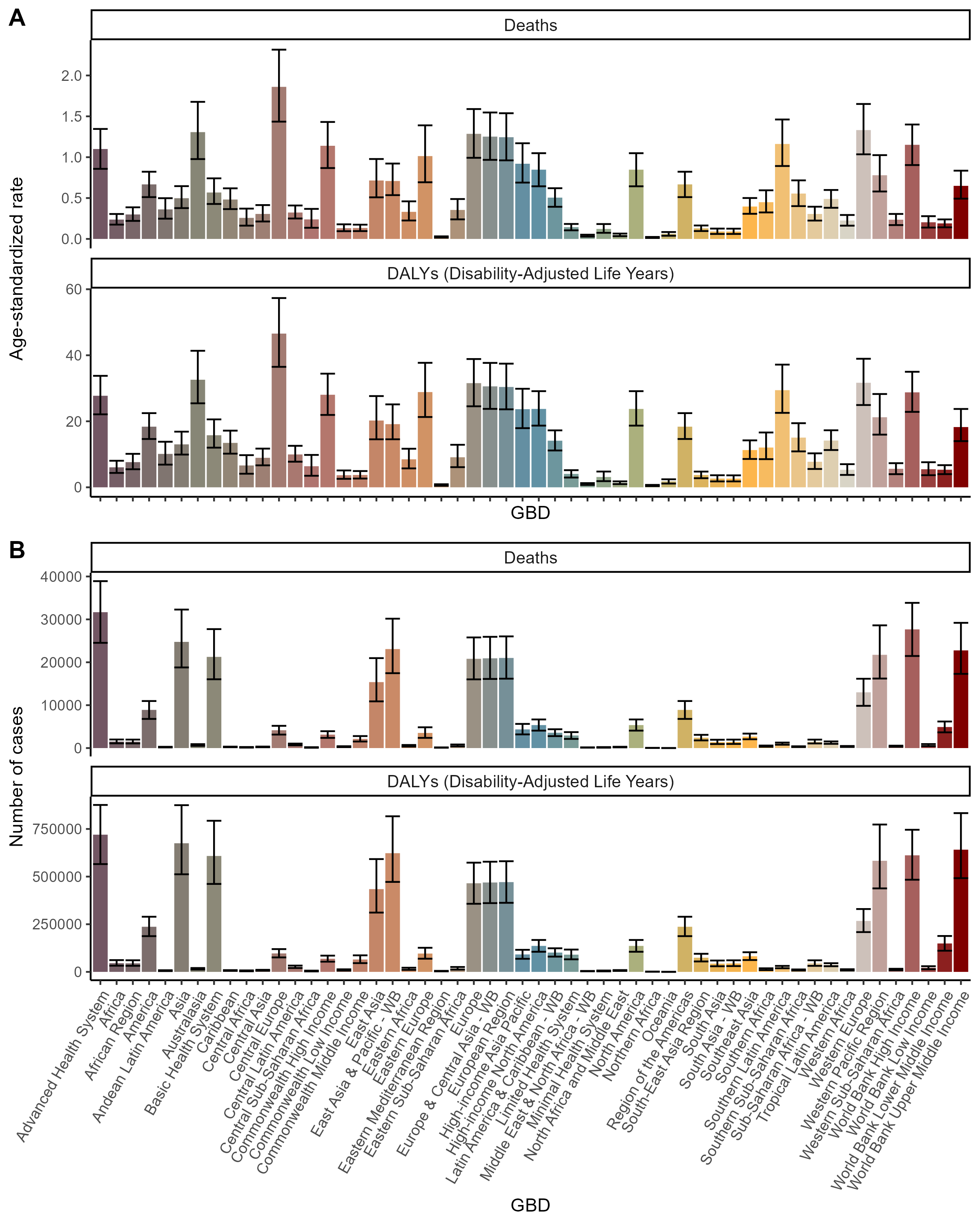

Supplement: Supplementary file 4 [file Image_4.TIF]

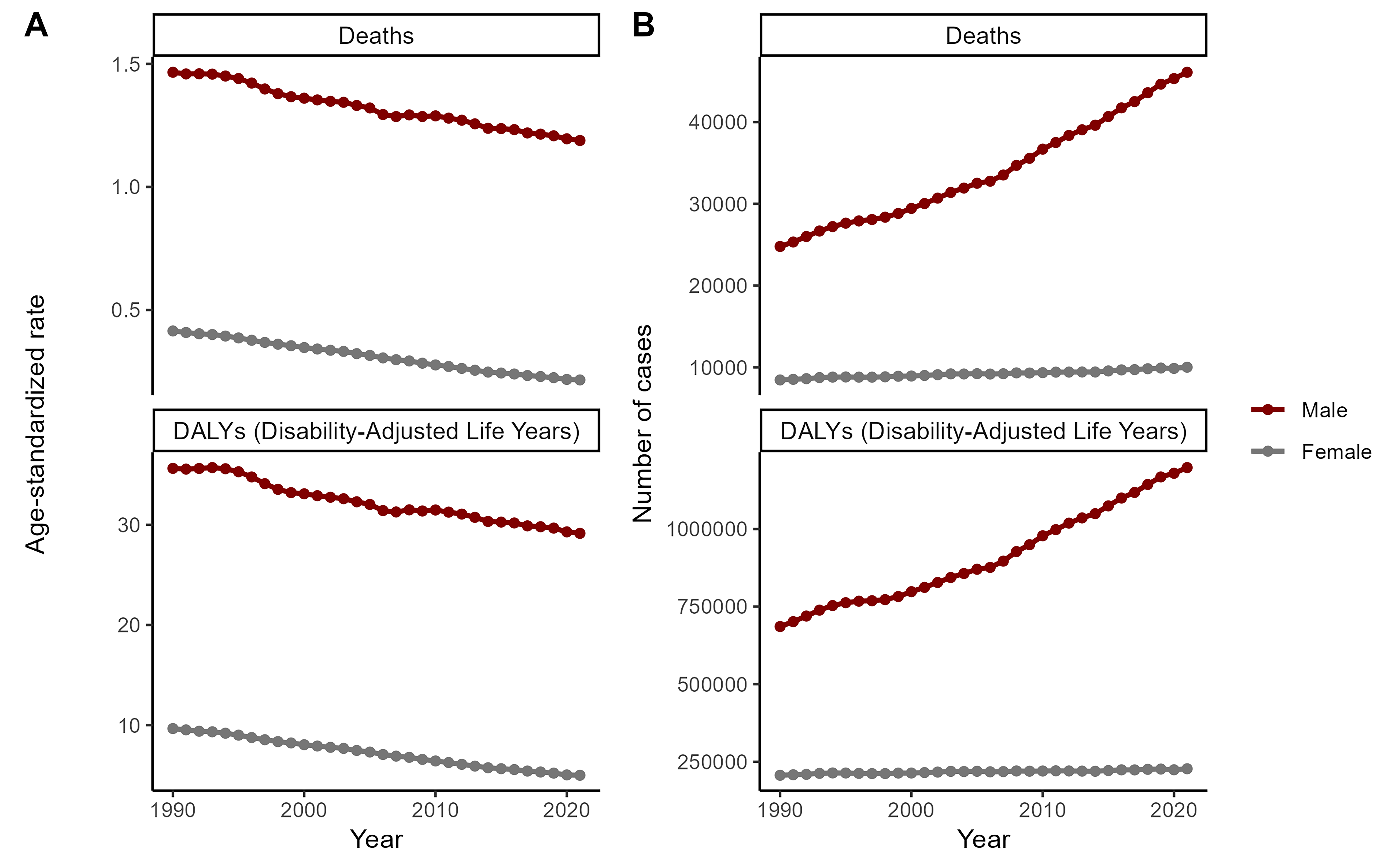

Supplement: Supplementary file 5 [file Image_5.TIF]

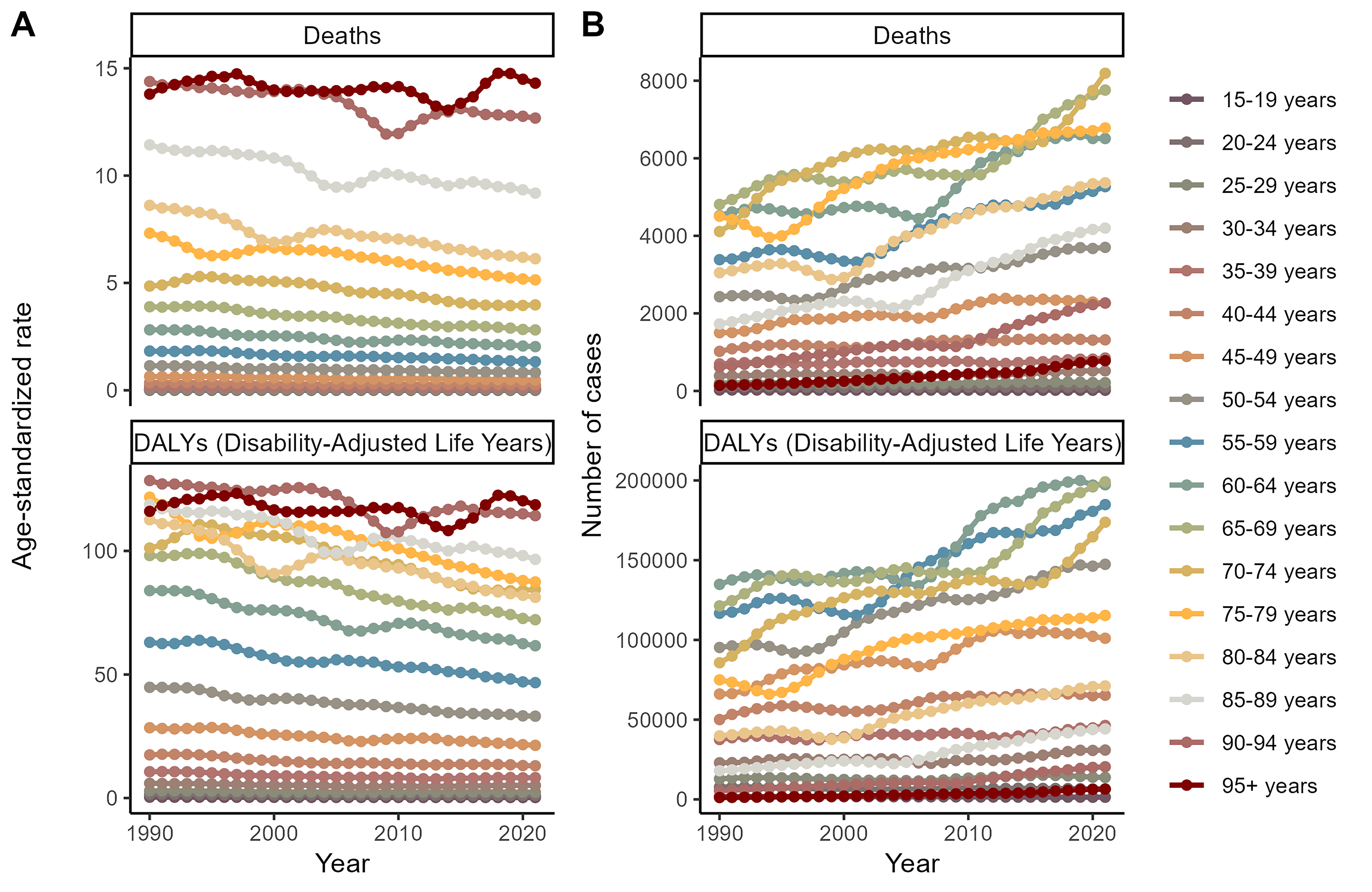

Supplement: Supplementary file 6 [file Image_6.TIF]

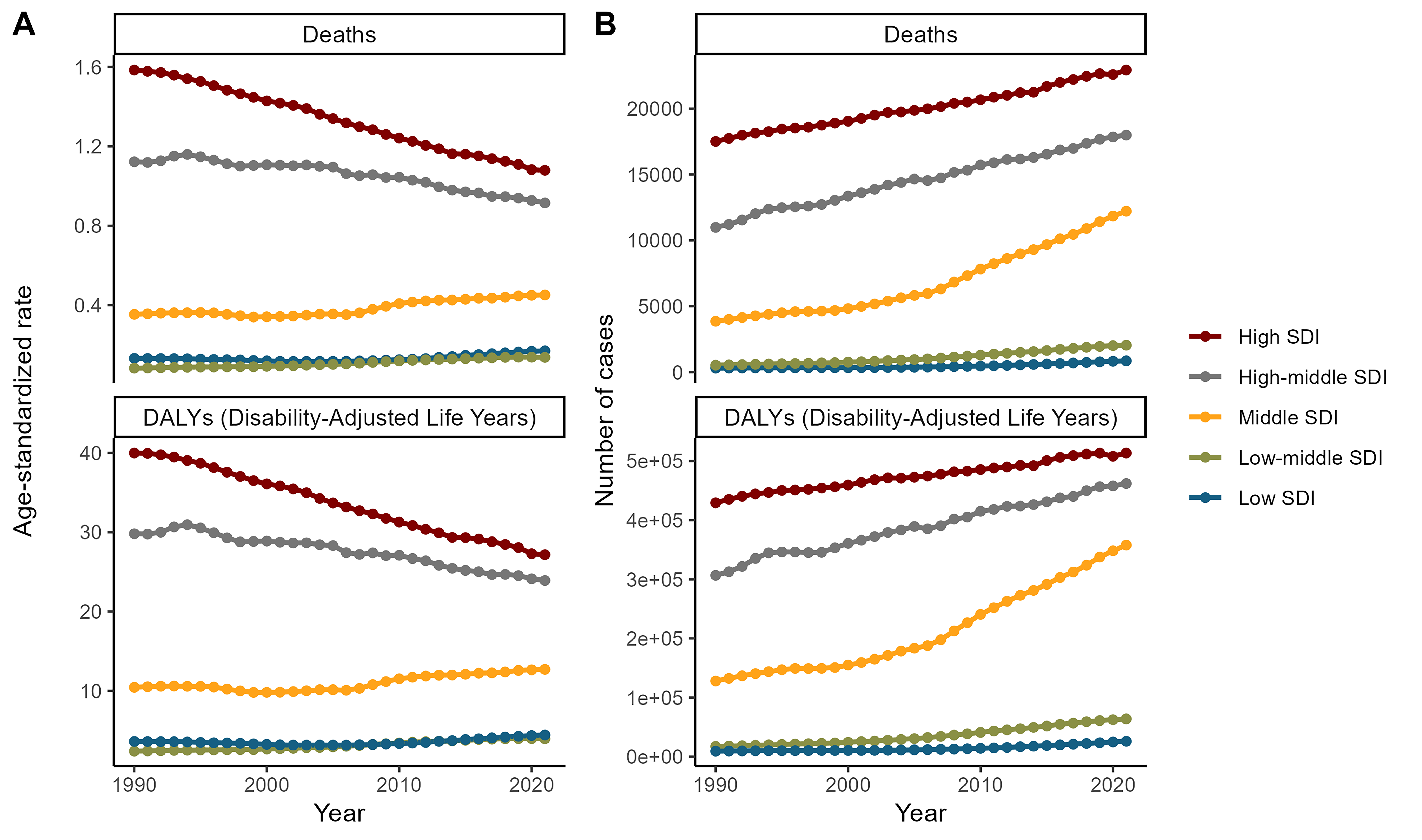

Supplement: Supplementary file 7 [file Image_7.TIF]

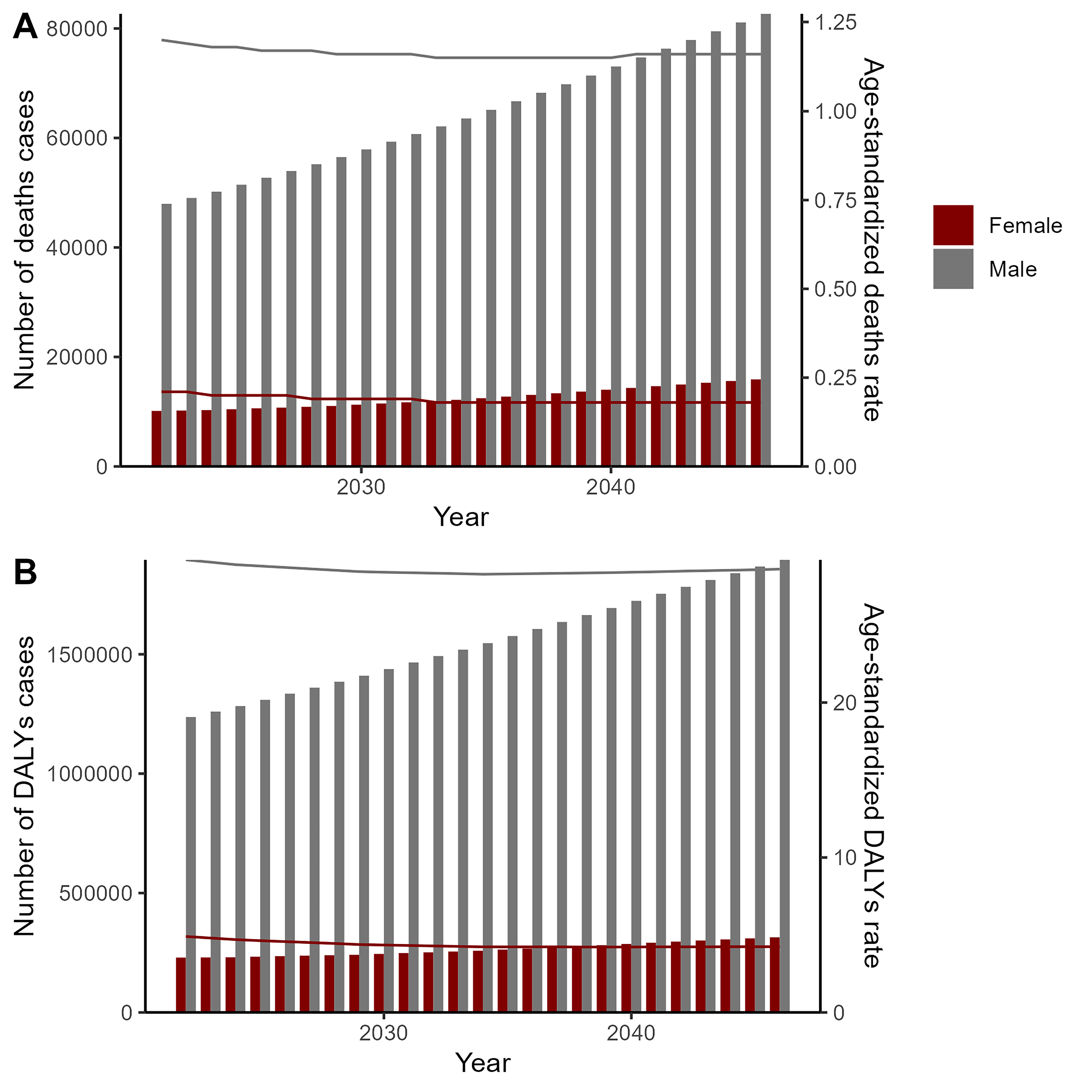

Supplement: Supplementary file 8 [file Image_8.TIF]

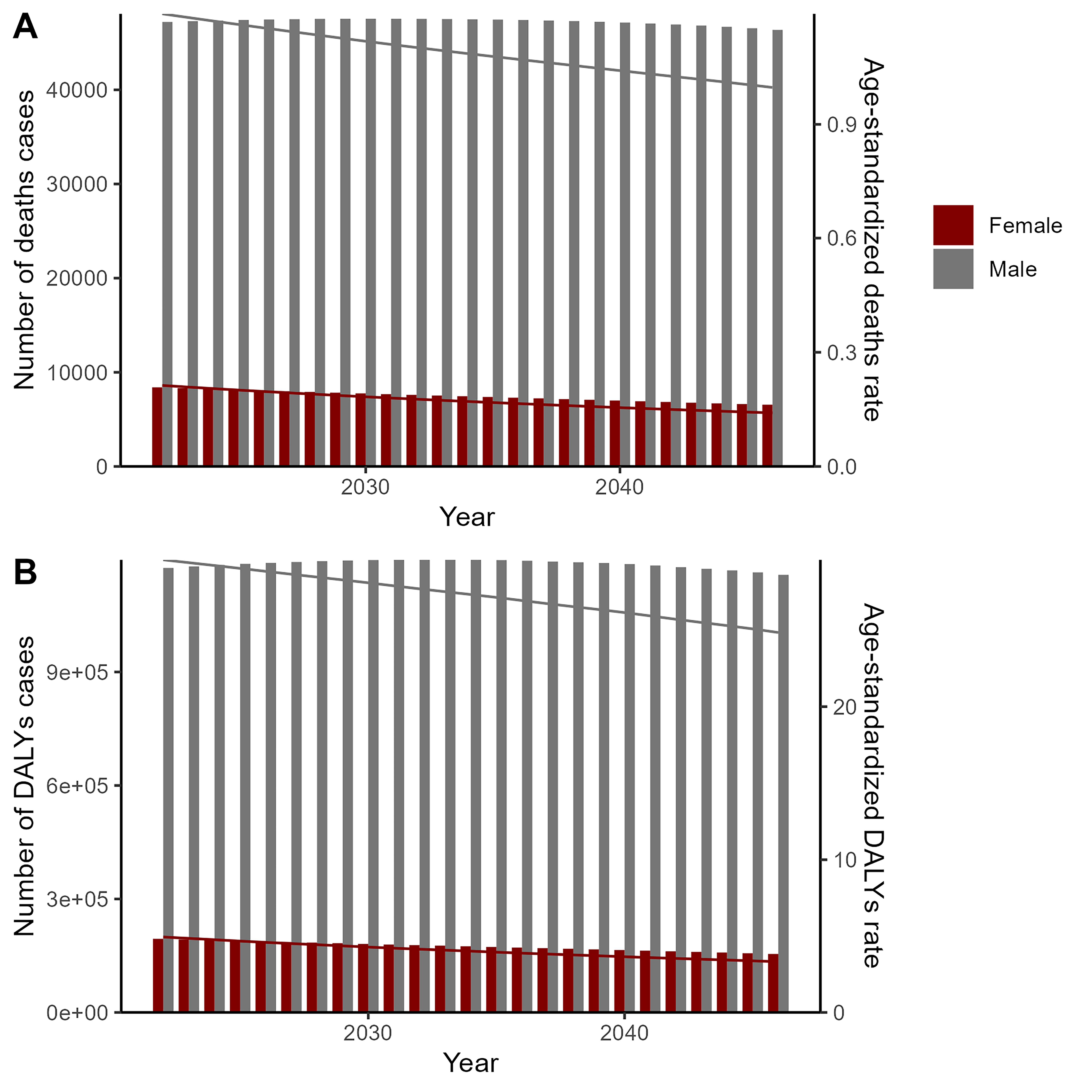

Supplement: Supplementary file 9 [file Image_9.TIF]
