# Supplementary material for: The global, regional, and national alcohol-related colorectal cancer burden and forecasted trends: results from the global burden of disease study 2021
Source: Front Nutr. 2024 Dec 24;11:1520852. doi: 10.3389/fnut.2024.1520852 (PMC11704491; doi:10.3389/fnut.2024.1520852)

Supplemental Table legend

Table S1. The predicted results in alcohol-related colorectal cancer-related numbers and age-standardized rates of deaths and DALYs by sex globally from 2022 to 2050 of the APC model. Abbreviations: DALYs, disability-adjusted-life-year; APC, Age-Period-Cohort .

Table S2. The predicted results in Alcohol-related colorectal cancerrelated numbers and age-standardized rates of deaths and DALYs by sex globally from 2022 to 2050 of the BAPC model. Abbreviations: DALYs, disability-adjusted-life-year; BAPC, Bayes age-period-cohort.

Supplemental Figure legend

Figure S1. Numbers and age-standardized rates of alcohol-related colorectal cancer-related deaths and DALYs for both sexes in 2021. Abbreviations: DALYs, disability-adjusted life years.

Figure S2. Numbers and age-standardized rates of alcohol-related colorectal cancer-related deaths and DALYs for different age groups in 2021. Abbreviations: DALYs, disability-adjusted life years.

Figure S3. Numbers and age-standardized rates of alcohol-related colorectal cancer-related deaths and DALYs for different SDI regions in 2021. Abbreviations: DALYs, disability-adjusted-life-years; SDI, socio-demographic index.

Figure S4. Numbers and age-standardized rates of alcohol-related colorectal cancer-related deaths and DALYs for different GBD regions in 2021. Abbreviations: DALYs, disability-adjusted life years; GBD, Global Burden of Disease.

Figure S5. Trends in the numbers and age-standardized rates of alcohol-related colorectal cancer-related deaths and DALYs globally by sexes from 1990 to 2021. Abbreviations: DALYs, disability-adjusted-life-years.

Figure S6. Trends in the numbers and age-standardized rates of alcohol-related colorectal cancer-related deaths and DALYs globally by age groups from 1990 to 2021. Abbreviations: DALYs, disability-adjusted-life-years.

Figure S7. Trends in the numbers and age-standardized rates of alcohol-related

colorectal cancer-related deaths and DALYs globally by SDI regions from 1990 to 2021. Abbreviations: DALYs, disability-adjusted-life-years; SDI, socio-demographic index.

Figure S8. The predicted results in alcohol-related colorectal cancer-related numbers and age-standardized rates of deaths and DALYs by sex globally from 2022 to 2046 of the APC model. Abbreviations: DALYs, disability-adjusted-life-year; APC, Age-Period-Cohort.

Figure S9. The predicted results in alcohol-related colorectal cancer-related numbers and age-standardized rates of deaths and DALYs by sex globally from 2022 to 2046 of the BAPC model. Abbreviations: DALYs, disability-adjusted-life-year; BAPC, Bayes age-period-cohort.

Figure S1. Numbers and age-standardized rates of alcohol-related colorectal cancer-related deaths and DALYs for both sexes in 2021. Abbreviations: DALYs, disability-adjusted life years.

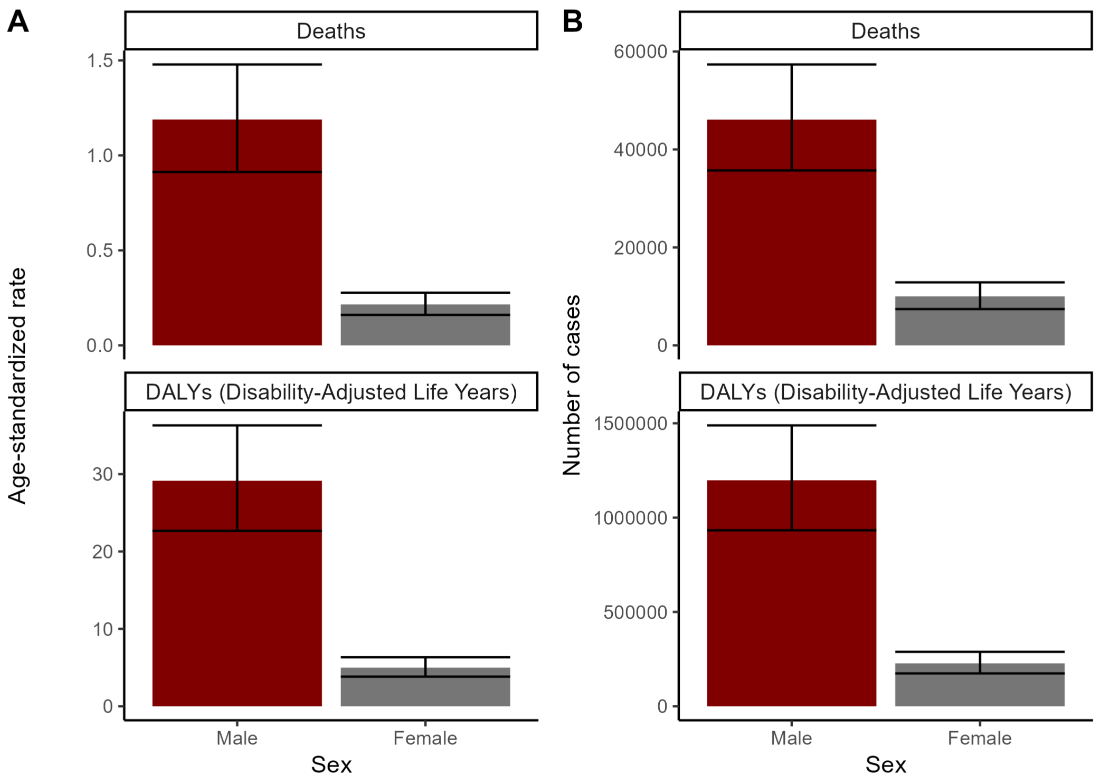

Figure S2. Numbers and age-standardized rates of alcohol-related colorectal cancer-related deaths and DALYs for different age groups in 2021. Abbreviations: DALYs, disability-adjusted life years.

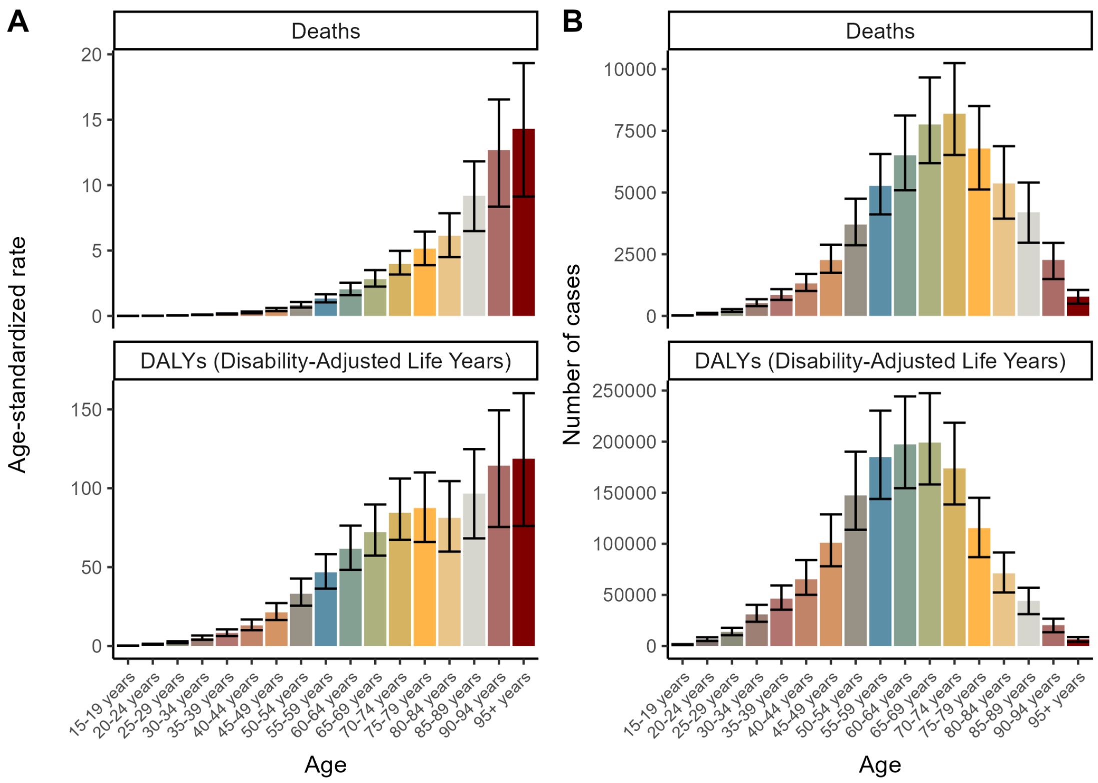

Figure S3. Numbers and age-standardized rates of alcohol-related colorectal cancer-related deaths and DALYs for different SDI regions in 2021. Abbreviations: DALYs, disability-adjusted-life-years; SDI, socio-demographic index.

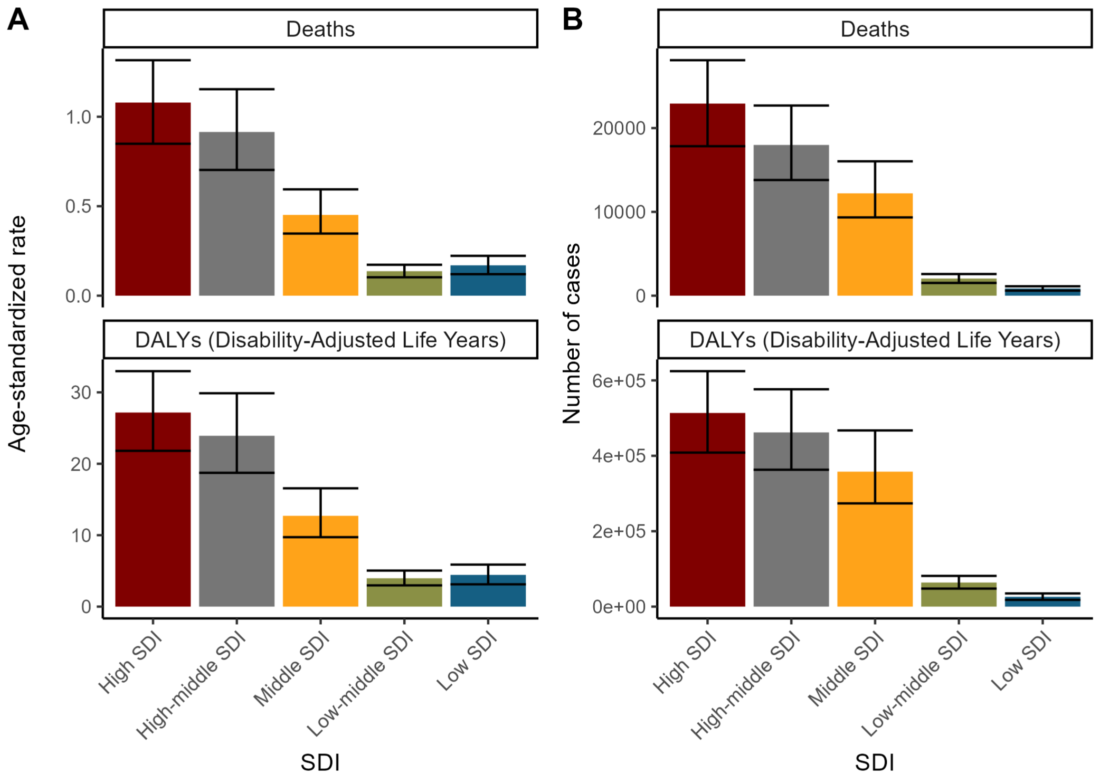

Figure S4. Numbers and age-standardized rates of alcohol-related colorectal cancer-related deaths and DALYs for different GBD regions in 2021. Abbreviations: DALYs, disability-adjusted life years; GBD, Global Burden of Disease.

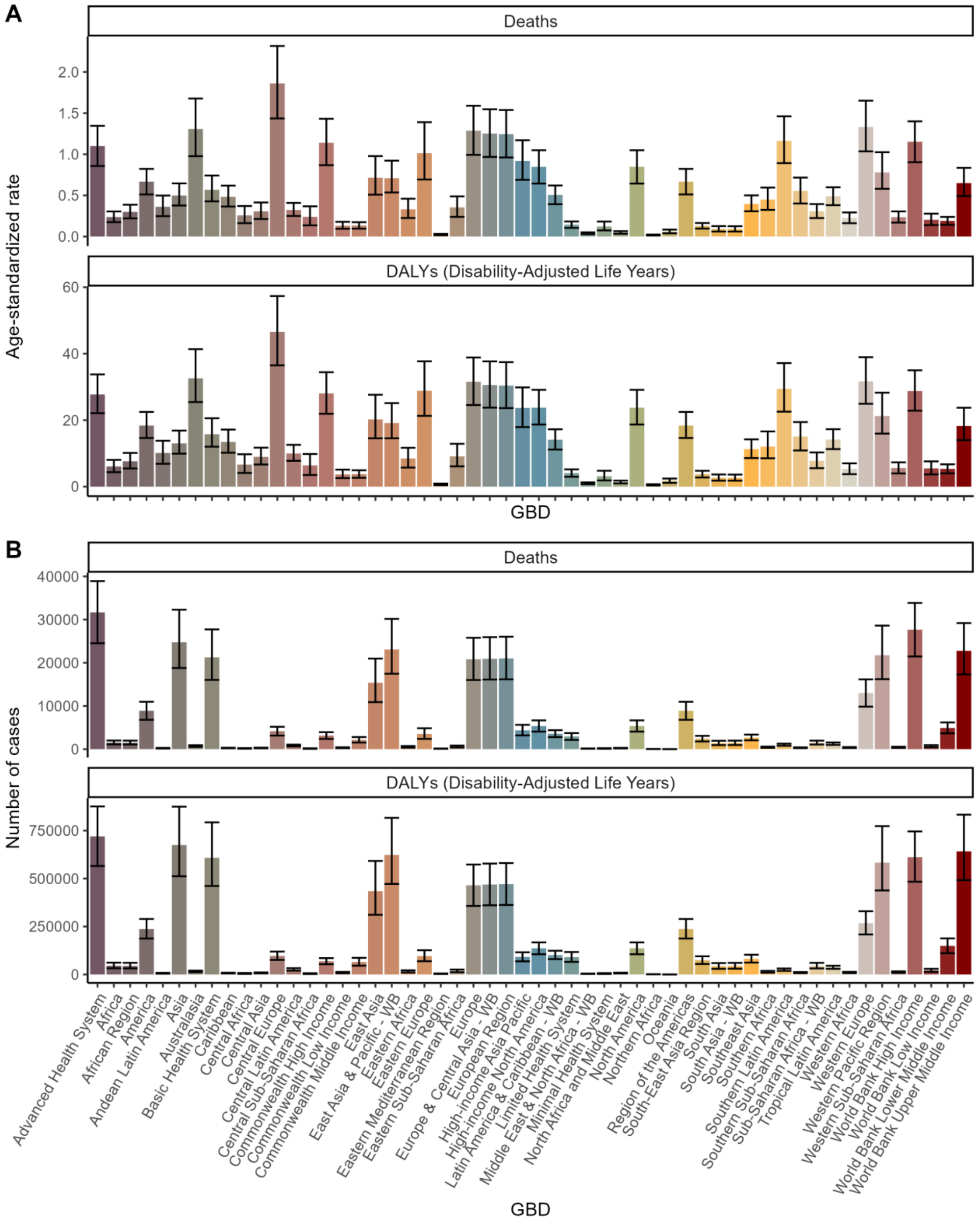

Figure S5. Trends in the numbers and age-standardized rates of alcohol-related colorectal cancer-related deaths and DALYs globally by sexes from 1990 to 2021. Abbreviations: DALYs, disability-adjusted-life-years.

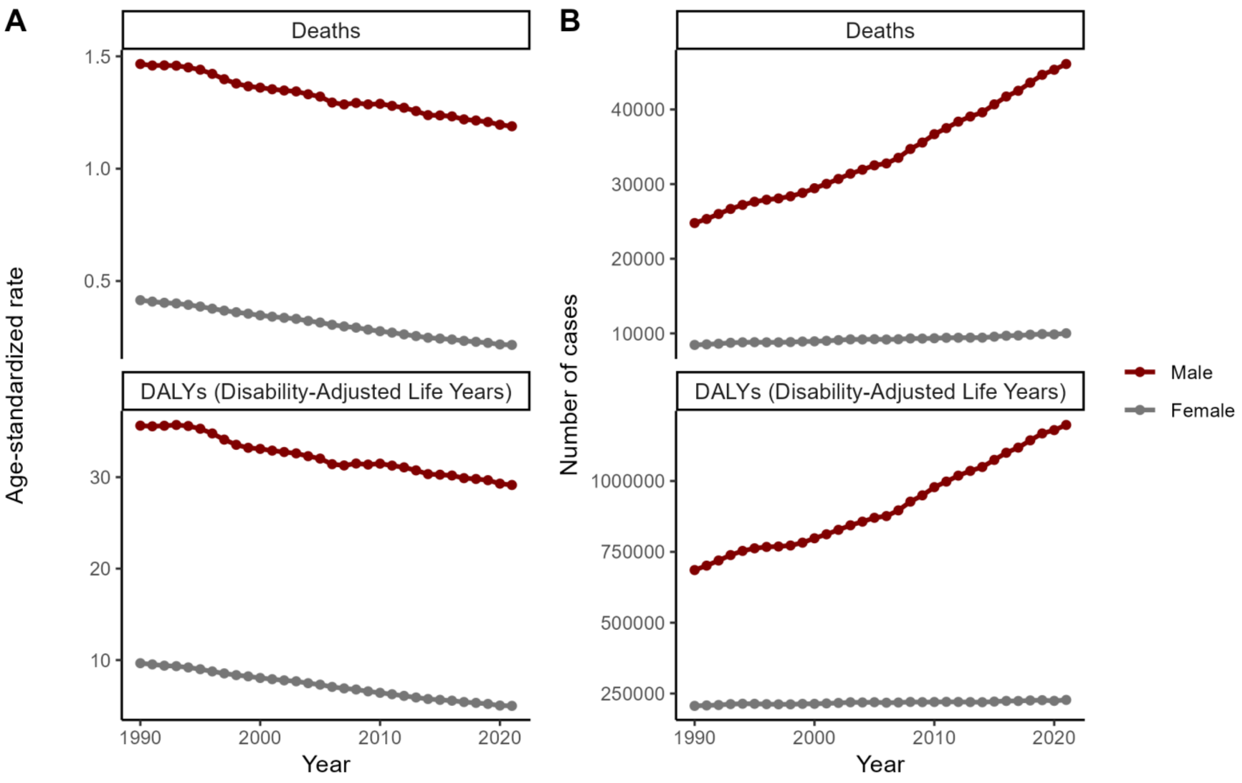

Figure S6. Trends in the numbers and age-standardized rates of alcohol-related colorectal cancer-related deaths and DALYs globally by age groups from 1990 to 2021. Abbreviations: DALYs, disability-adjusted-life-years.

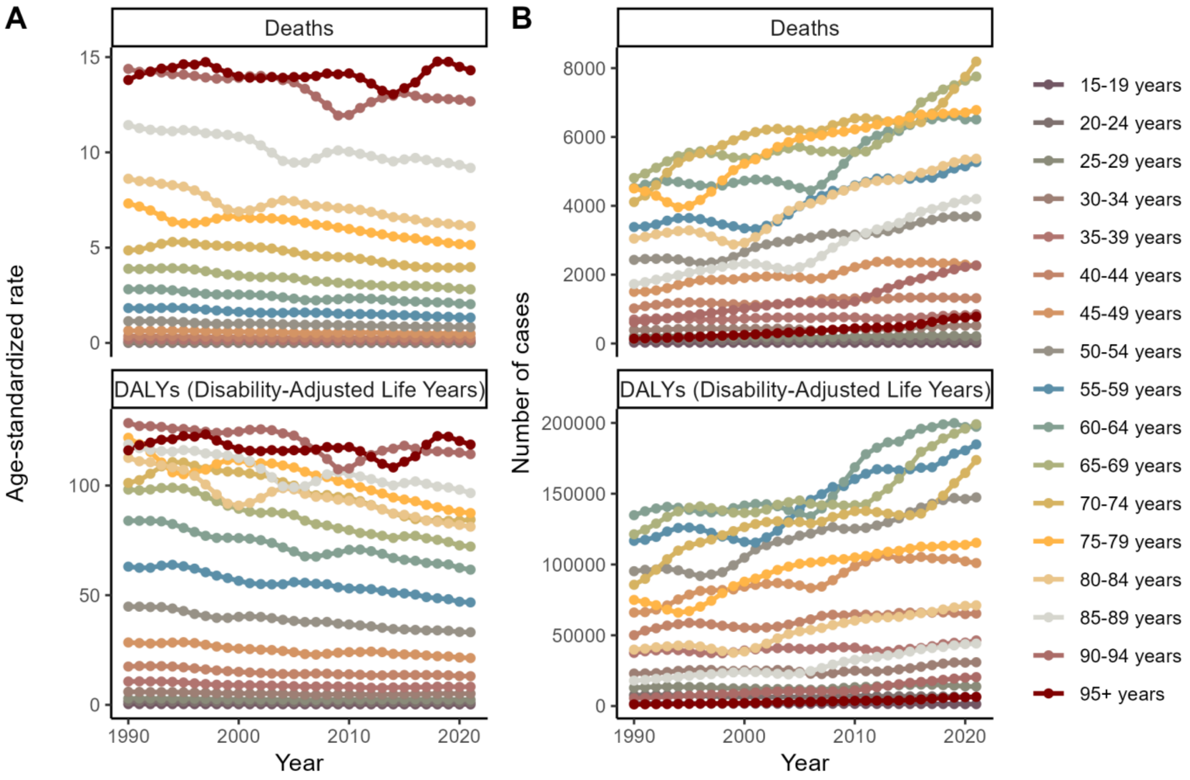

Figure S7. Trends in the numbers and age-standardized rates of alcohol-related colorectal cancer-related deaths and DALYs globally by SDI regions from 1990 to 2021. Abbreviations: DALYs, disability-adjusted-life-years; SDI, socio-demographic index.

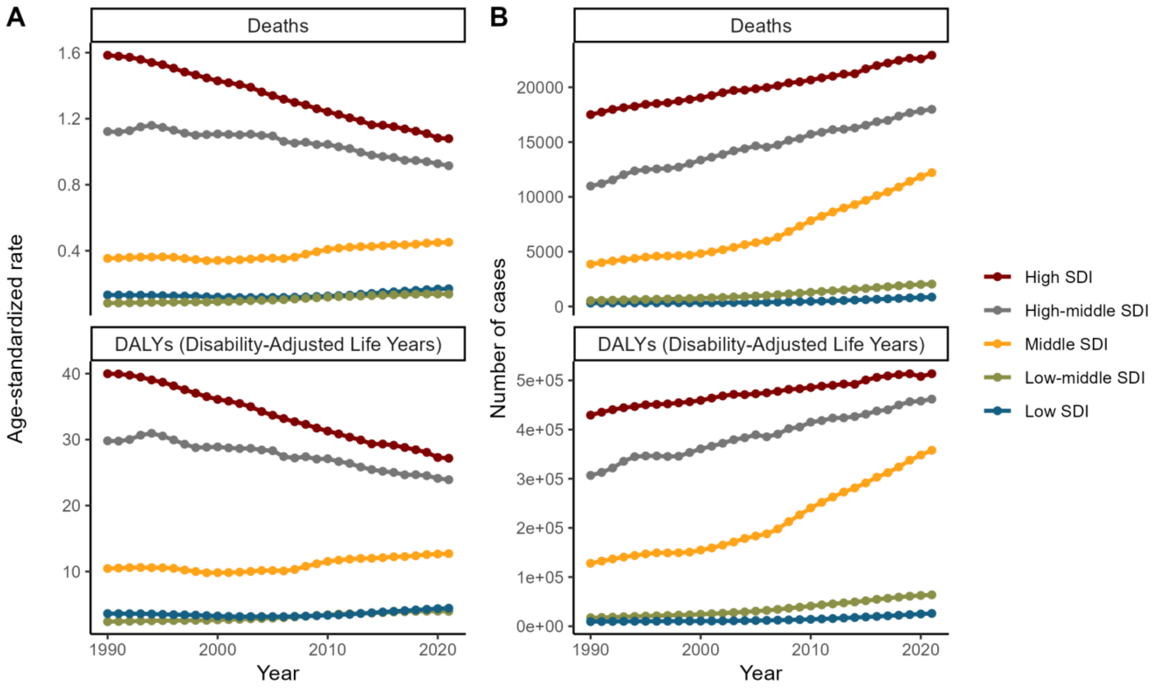

Figure S8. The predicted results in alcohol-related colorectal cancer-related numbers and age-standardized rates of deaths and DALYs by sex globally from 2022 to 2046 of the APC model. Abbreviations: DALYs, disability-adjusted-life-year; APC, Age-Period-Cohort.

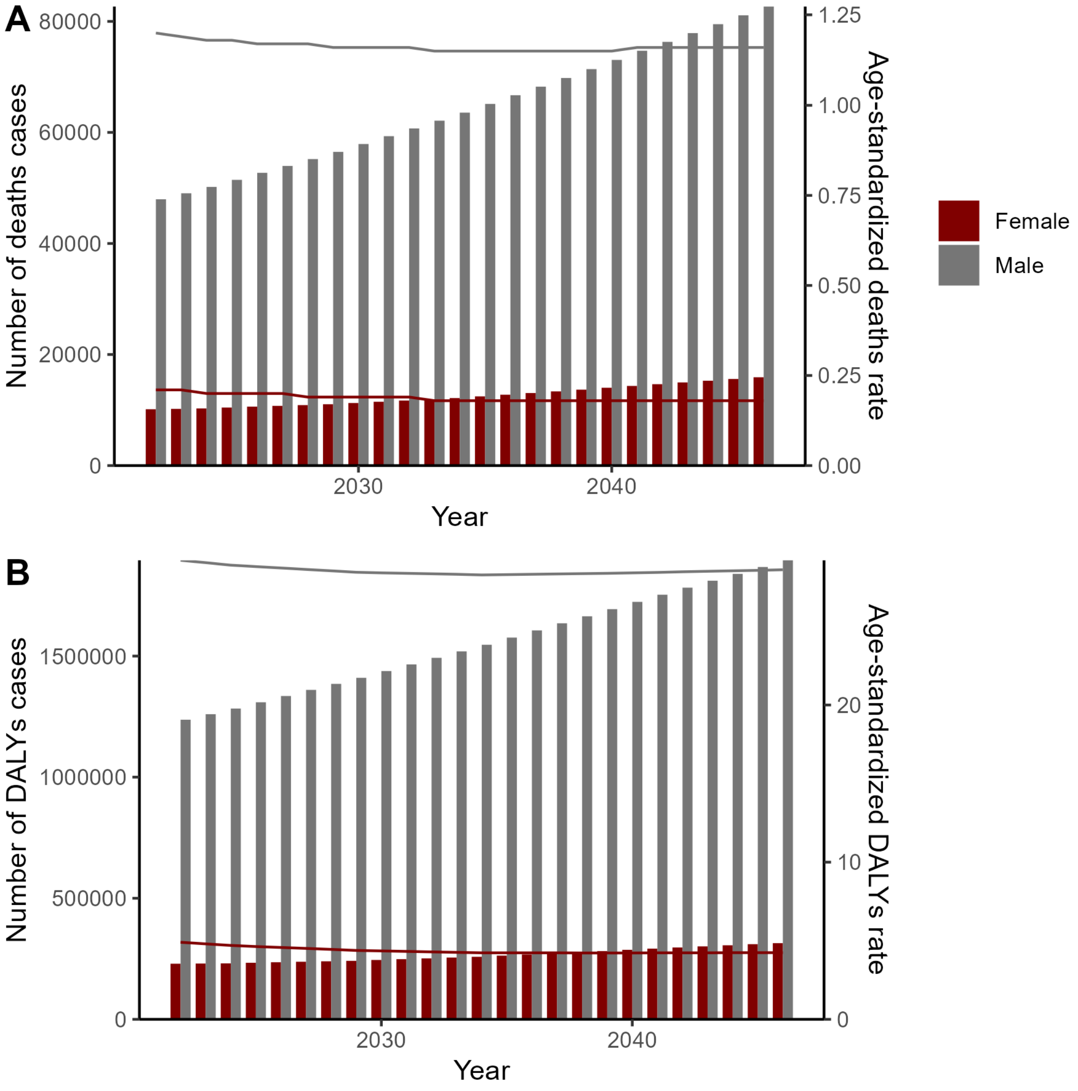

Figure S9. The predicted results in alcohol-related colorectal cancer-related numbers and age-standardized rates of deaths and DALYs by sex globally from 2022 to 2046 of the BAPC model. Abbreviations: DALYs, disability-adjusted-life-year; BAPC, Bayes age-period-cohort.

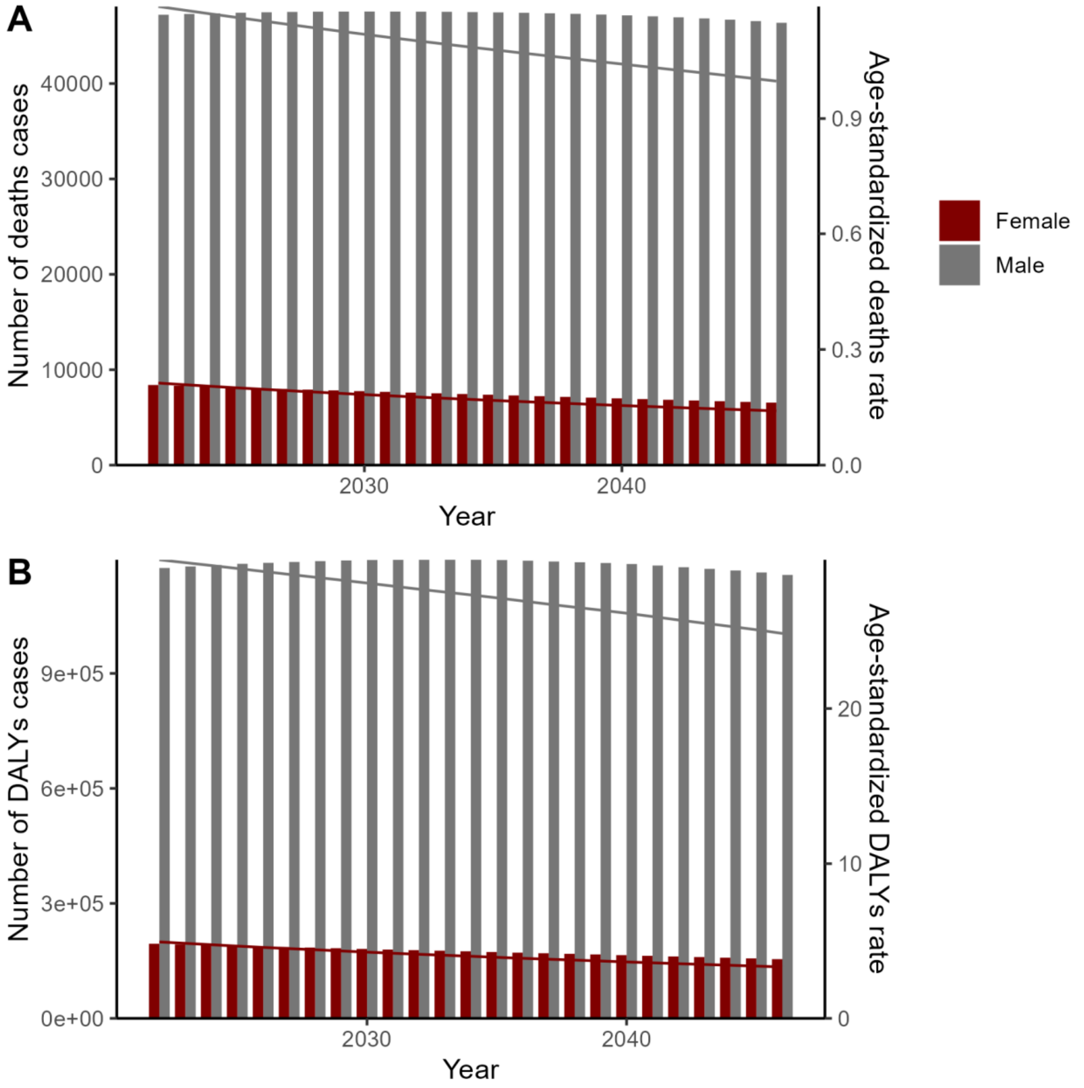

Supplement: Supplementary file 11 [file Table_1.pdf]
