# Supplementary material for: The global, regional, and national alcohol-related colorectal cancer burden and forecasted trends: results from the global burden of disease study 2021
Source: Front Nutr. 2024 Dec 24;11:1520852. doi: 10.3389/fnut.2024.1520852 (PMC11704491; doi:10.3389/fnut.2024.1520852)
Supplement: Supplementary file 13 [file Table_3.docx]

**Supplementary Table 3.** The number of deaths cases and the age-standardized deaths rate of alcohol-related colorectal cancer in 1990 and 2021, and its trends from 1990 to 2021 globally.

|  | Number of deaths cases (95% UI) in 1990 | The age-standardized deaths rate/100000 (95% UI) in 1990 | Number of deaths cases (95% UI) in 2021 | The age-standardized deaths rate/100000 (95% UI) in 2021 | EAPC (95% CI) |  |
| --- | --- | --- | --- | --- | --- | --- |
| Global | 33239 (25537-41330) | 0.88 (0.66-1.09) | 56102 (43738-69936) | 0.66 (0.51-0.82) | -0.96 (-0.98--0.93) |  |
| Sex |  |  |  |  |  |  |
| Female | 8457 (6303-10861) | 0.41 (0.3-0.54) | 10012 (7409-12863) | 0.22 (0.16-0.28) | -2.19 (-2.25--2.14) |  |
| Male | 24783 (18931-30561) | 1.47 (1.1-1.83) | 46090 (35716-57352) | 1.19 (0.91-1.48) | -0.69 (-0.72--0.67) |  |
| Age |  |  |  |  |  |  |
| 15-19 years | 25 (17-32) | 0 (0-0.01) | 18 (13-25) | 0 (0-0) | -1.52 (-1.63--1.41) |  |
| 20-24 years | 113 (81-145) | 0.02 (0.02-0.03) | 95 (72-124) | 0.02 (0.01-0.02) | -1.15 (-1.35--0.95) |  |
| 25-29 years | 201 (150-258) | 0.05 (0.03-0.06) | 214 (164-276) | 0.04 (0.03-0.05) | -0.64 (-0.85--0.44) |  |
| 30-34 years | 390 (292-490) | 0.1 (0.08-0.13) | 520 (398-677) | 0.09 (0.07-0.11) | -0.67 (-0.87--0.46) |  |
| 35-39 years | 696 (536-872) | 0.2 (0.15-0.25) | 849 (650-1085) | 0.15 (0.12-0.19) | -1.01 (-1.18--0.85) |  |
| 40-44 years | 1020 (789-1260) | 0.36 (0.28-0.44) | 1317 (1011-1701) | 0.26 (0.2-0.34) | -1.04 (-1.18--0.9) |  |
| 45-49 years | 1501 (1172-1841) | 0.65 (0.5-0.79) | 2265 (1748-2884) | 0.48 (0.37-0.61) | -0.95 (-1.06--0.85) |  |
| 50-54 years | 2430 (1946-2949) | 1.14 (0.92-1.39) | 3702 (2865-4748) | 0.83 (0.64-1.07) | -1.06 (-1.12--1) |  |
| 55-59 years | 3384 (2704-4100) | 1.83 (1.46-2.21) | 5268 (4113-6560) | 1.33 (1.04-1.66) | -1.02 (-1.1--0.95) |  |
| 60-64 years | 4522 (3632-5526) | 2.82 (2.26-3.44) | 6509 (5092-8120) | 2.03 (1.59-2.54) | -1.02 (-1.11--0.93) |  |
| 65-69 years | 4809 (3670-5928) | 3.89 (2.97-4.8) | 7758 (6189-9661) | 2.81 (2.24-3.5) | -1.17 (-1.23--1.1) |  |
| 70-74 years | 4111 (3061-5182) | 4.86 (3.62-6.12) | 8192 (6520-10243) | 3.98 (3.17-4.98) | -1.01 (-1.15--0.88) |  |
| 75-79 years | 4505 (3154-5758) | 7.32 (5.12-9.35) | 6782 (5122-8502) | 5.14 (3.88-6.45) | -0.94 (-1.07--0.82) |  |
| 80-84 years | 3048 (1942-3988) | 8.61 (5.49-11.27) | 5368 (3940-6877) | 6.13 (4.5-7.85) | -0.99 (-1.12--0.85) |  |
| 85-89 years | 1728 (1029-2308) | 11.43 (6.81-15.28) | 4199 (2966-5403) | 9.18 (6.49-11.82) | -0.68 (-0.79--0.57) |  |
| 90-94 years | 616 (334-858) | 14.38 (7.8-20.02) | 2268 (1494-2960) | 12.68 (8.35-16.55) | -0.47 (-0.59--0.35) |  |
| 95+ years | 140 (68-204) | 13.8 (6.63-20.02) | 780 (497-1054) | 14.31 (9.13-19.33) | -0.06 (-0.18-0.05) |  |
| SDI region |  |  |  |  |  |  |
| High-middle SDI | 10984 (8487-13571) | 1.12 (0.85-1.39) | 17985 (13802-22690) | 0.91 (0.7-1.15) | -0.72 (-0.8--0.64) |  |
| High SDI | 17506 (13039-22013) | 1.58 (1.18-1.99) | 22924 (17836-28098) | 1.08 (0.85-1.32) | -1.31 (-1.35--1.28) |  |
| Low-middle SDI | 531 (371-700) | 0.08 (0.06-0.11) | 2042 (1519-2581) | 0.14 (0.1-0.17) | 1.92 (1.8-2.04) |  |
| Low SDI | 305 (181-421) | 0.13 (0.08-0.18) | 854 (601-1123) | 0.17 (0.12-0.22) | 0.79 (0.45-1.13) |  |
| Middle SDI | 3859 (2841-4907) | 0.35 (0.26-0.45) | 12211 (9341-16035) | 0.45 (0.35-0.59) | 0.93 (0.74-1.12) |  |
| GBD region |  |  |  |  |  |  |
| Advanced Health System | 24652 (18244-30935) | 1.51 (1.12-1.9) | 31678 (24529-38912) | 1.1 (0.86-1.35) | -1.13 (-1.22--1.04) |  |
| Africa | 531 (339-697) | 0.19 (0.13-0.25) | 1517 (1100-1997) | 0.24 (0.17-0.31) | 0.7 (0.63-0.77) |  |
| African Region | 518 (330-680) | 0.24 (0.16-0.31) | 1494 (1081-1976) | 0.3 (0.22-0.39) | 0.76 (0.68-0.83) |  |
| America | 4946 (3481-6581) | 0.81 (0.57-1.08) | 8898 (6797-10967) | 0.67 (0.51-0.82) | -0.47 (-0.52--0.42) |  |
| Andean Latin America | 60 (39-83) | 0.28 (0.19-0.39) | 216 (148-297) | 0.36 (0.25-0.5) | 1.24 (1.02-1.46) |  |
| Asia | 9765 (7377-12039) | 0.49 (0.37-0.6) | 24756 (18796-32288) | 0.5 (0.38-0.65) | 0.08 (-0.07-0.22) |  |
| Australasia | 500 (312-687) | 2.15 (1.36-2.95) | 710 (522-913) | 1.31 (0.98-1.68) | -1.7 (-1.76--1.64) |  |
| Basic Health System | 7673 (5723-9640) | 0.5 (0.38-0.63) | 21257 (16037-27731) | 0.57 (0.43-0.74) | 0.54 (0.36-0.73) |  |
| Caribbean | 106 (79-132) | 0.41 (0.3-0.51) | 259 (196-333) | 0.48 (0.36-0.62) | 0.84 (0.76-0.93) |  |
| Central Africa | 64 (36-92) | 0.23 (0.14-0.33) | 168 (104-246) | 0.26 (0.16-0.37) | 0.72 (0.14-1.31) |  |
| Central Asia | 200 (139-262) | 0.4 (0.27-0.53) | 262 (190-349) | 0.31 (0.21-0.41) | -0.46 (-0.65--0.26) |  |
| Central Europe | 2450 (1807-3090) | 1.64 (1.19-2.07) | 4154 (3145-5184) | 1.86 (1.43-2.32) | 0.38 (0.28-0.48) |  |
| Central Latin America | 211 (168-263) | 0.24 (0.19-0.3) | 826 (639-1045) | 0.32 (0.25-0.41) | 0.88 (0.71-1.06) |  |
| Central Sub-Saharan Africa | 47 (21-72) | 0.21 (0.1-0.32) | 133 (73-205) | 0.24 (0.14-0.37) | 1.04 (0.36-1.73) |  |
| Commonwealth High Income | 2494 (1701-3260) | 1.66 (1.15-2.15) | 3135 (2376-3933) | 1.14 (0.87-1.43) | -1.23 (-1.31--1.14) |  |
| Commonwealth Low Income | 104 (64-142) | 0.12 (0.08-0.16) | 312 (222-430) | 0.13 (0.09-0.18) | 0.16 (0.07-0.26) |  |
| Commonwealth Middle Income | 593 (377-792) | 0.09 (0.06-0.12) | 2138 (1527-2796) | 0.13 (0.1-0.17) | 1.2 (1.08-1.32) |  |
| East Asia | 5786 (4126-7512) | 0.65 (0.46-0.84) | 15377 (10887-20969) | 0.71 (0.51-0.98) | 0.43 (0.17-0.69) |  |
| East Asia & Pacific - WB | 9268 (6952-11518) | 0.7 (0.53-0.87) | 23081 (17458-30164) | 0.71 (0.54-0.92) | -0.02 (-0.17-0.14) |  |
| Eastern Africa | 178 (99-253) | 0.25 (0.14-0.35) | 522 (351-719) | 0.33 (0.23-0.46) | 0.79 (0.5-1.09) |  |
| Eastern Europe | 3001 (2012-3839) | 1.06 (0.71-1.35) | 3528 (2378-4843) | 1.01 (0.69-1.39) | -0.33 (-0.68-0.03) |  |
| Eastern Mediterranean Region | 35 (24-48) | 0.02 (0.01-0.02) | 114 (79-155) | 0.02 (0.02-0.03) | 1.25 (1.02-1.48) |  |
| Eastern Sub-Saharan Africa | 195 (106-275) | 0.25 (0.14-0.36) | 590 (396-833) | 0.35 (0.24-0.49) | 0.97 (0.67-1.28) |  |
| Europe | 17923 (13016-22416) | 1.73 (1.26-2.17) | 20810 (16015-25791) | 1.29 (0.99-1.59) | -1.07 (-1.19--0.95) |  |
| Europe & Central Asia - WB | 18042 (13126-22563) | 1.7 (1.24-2.12) | 20922 (16115-25936) | 1.25 (0.97-1.55) | -0.75 (-1.58-0.09) |  |
| European Region | 18079 (13153-22611) | 1.69 (1.23-2.11) | 21007 (16175-26035) | 1.24 (0.96-1.54) | -1.09 (-1.21--0.97) |  |
| High-income Asia Pacific | 2554 (1952-3202) | 1.28 (0.98-1.61) | 4351 (3186-5633) | 0.92 (0.69-1.17) | -1.36 (-1.44--1.27) |  |
| High-income North America | 3538 (2269-4924) | 1.02 (0.67-1.41) | 5359 (4067-6670) | 0.85 (0.64-1.05) | -0.46 (-0.52--0.4) |  |
| Latin America & Caribbean - WB | 1429 (1125-1751) | 0.54 (0.43-0.66) | 3575 (2786-4387) | 0.51 (0.39-0.62) | -0.06 (-0.13-0) |  |
| Limited Health System | 795 (510-1067) | 0.1 (0.06-0.13) | 2933 (2135-3719) | 0.14 (0.11-0.18) | 1.47 (1.27-1.67) |  |
| Middle East & North Africa - WB | 37 (25-52) | 0.03 (0.02-0.04) | 128 (94-166) | 0.04 (0.03-0.05) | 1.19 (0.98-1.4) |  |
| Minimal Health System | 65 (36-93) | 0.11 (0.06-0.16) | 149 (89-227) | 0.12 (0.07-0.18) | 0.46 (-0.04-0.96) |  |
| North Africa and Middle East | 98 (68-132) | 0.05 (0.04-0.07) | 230 (163-304) | 0.05 (0.03-0.07) | -0.38 (-0.56--0.2) |  |
| North America | 3538 (2270-4924) | 1.02 (0.67-1.41) | 5359 (4067-6670) | 0.85 (0.64-1.05) | -0.46 (-0.53--0.4) |  |
| Northern Africa | 7 (5-10) | 0.01 (0.01-0.01) | 29 (19-40) | 0.02 (0.01-0.02) | 2.03 (1.86-2.21) |  |
| Oceania | 2 (1-3) | 0.06 (0.04-0.09) | 5 (3-7) | 0.06 (0.04-0.08) | 0.31 (0.01-0.6) |  |
| Region of the Americas | 4946 (3481-6581) | 0.81 (0.57-1.08) | 8898 (6797-10967) | 0.67 (0.51-0.82) | -0.47 (-0.52--0.42) |  |
| South-East Asia Region | 599 (401-795) | 0.08 (0.05-0.11) | 2408 (1787-3087) | 0.13 (0.1-0.16) | 1.65 (1.51-1.78) |  |
| South Asia | 316 (159-473) | 0.05 (0.03-0.08) | 1394 (939-1941) | 0.09 (0.06-0.13) | 2.15 (1.9-2.4) |  |
| South Asia - WB | 325 (168-484) | 0.05 (0.03-0.08) | 1424 (960-1975) | 0.09 (0.06-0.13) | 2.13 (1.9-2.37) |  |
| Southeast Asia | 435 (318-564) | 0.16 (0.12-0.2) | 2670 (2048-3373) | 0.4 (0.31-0.5) | 3.29 (3.16-3.42) |  |
| Southern Africa | 151 (104-200) | 0.35 (0.25-0.47) | 431 (307-589) | 0.45 (0.32-0.6) | 0.78 (0.58-0.97) |  |
| Southern Latin America | 748 (582-928) | 1.63 (1.28-2.03) | 1016 (778-1278) | 1.16 (0.89-1.46) | -0.69 (-0.82--0.56) |  |
| Southern Sub-Saharan Africa | 129 (93-173) | 0.47 (0.34-0.64) | 323 (235-416) | 0.56 (0.4-0.72) | 0.41 (0.2-0.62) |  |
| Sub-Saharan Africa - WB | 524 (334-688) | 0.24 (0.16-0.32) | 1488 (1075-1970) | 0.31 (0.22-0.39) | 0.66 (0.51-0.81) |  |
| Tropical Latin America | 308 (236-383) | 0.32 (0.25-0.41) | 1267 (989-1551) | 0.49 (0.38-0.6) | 1.31 (1.01-1.62) |  |
| Western Africa | 130 (86-177) | 0.17 (0.11-0.23) | 367 (260-485) | 0.23 (0.16-0.29) | 0.96 (0.87-1.06) |  |
| Western Europe | 12411 (9017-15472) | 2.13 (1.57-2.65) | 12999 (9847-16162) | 1.33 (1.04-1.65) | -1.6 (-1.67--1.53) |  |
| Western Pacific Region | 8844 (6611-11031) | 0.78 (0.59-0.98) | 21741 (16208-28609) | 0.78 (0.58-1.03) | 0 (-0.16-0.16) |  |
| Western Sub-Saharan Africa | 143 (97-196) | 0.17 (0.11-0.23) | 432 (307-562) | 0.24 (0.17-0.31) | 1.2 (1.1-1.29) |  |
| World Bank High Income | 21418 (15903-26861) | 1.66 (1.24-2.08) | 27666 (21462-33852) | 1.15 (0.9-1.4) | -1.26 (-1.31--1.21) |  |
| World Bank Low Income | 281 (174-396) | 0.18 (0.12-0.26) | 674 (453-931) | 0.2 (0.14-0.28) | 0.29 (-0.08-0.66) |  |
| World Bank Lower Middle Income | 1684 (1214-2171) | 0.16 (0.11-0.2) | 4907 (3664-6192) | 0.19 (0.14-0.24) | 0.84 (0.75-0.92) |  |
| World Bank Upper Middle Income | 9803 (7535-12210) | 0.64 (0.49-0.8) | 22770 (17296-29194) | 0.65 (0.49-0.84) | 0.06 (-0.03-0.14) |  |
| Country |  |  |  |  |  |  |
| Afghanistan | 0 (0-0) | 0 (0-0) | 0 (0-1) | 0 (0-0.01) | 0 (0-0) |  |
| Albania | 3 (1-5) | 0.14 (0.07-0.23) | 14 (9-21) | 0.32 (0.21-0.48) | 3.12 (2.34-3.91) |  |
| American Samoa | 0 (0-0) | 0.05 (0-0.14) | 0 (0-0) | 0.04 (0.01-0.1) | 0 (0-0) |  |
| Angola | 8 (3-13) | 0.19 (0.08-0.31) | 48 (28-69) | 0.39 (0.24-0.56) | 0 (0-0) |  |
| Antigua and Barbuda | 0 (0-0) | 0.16 (0.06-0.27) | 0 (0-1) | 0.47 (0.34-0.6) | 3.5 (2.94-4.08) |  |
| Argentine Republic | 599 (463-739) | 1.87 (1.45-2.32) | 679 (521-867) | 1.21 (0.93-1.55) | -0.88 (-1.08--0.69) |  |
| Armenia | 9 (5-13) | 0.31 (0.18-0.44) | 17 (11-23) | 0.39 (0.25-0.53) | 1.2 (0.87-1.52) |  |
| Australia | 391 (242-540) | 2.02 (1.26-2.78) | 572 (421-739) | 1.25 (0.93-1.61) | -1.66 (-1.74--1.59) |  |
| Austria | 283 (210-361) | 2.37 (1.76-3.03) | 198 (151-252) | 1.05 (0.81-1.32) | -2.59 (-2.65--2.54) |  |
| Azerbaijan | 20 (13-28) | 0.37 (0.24-0.51) | 32 (21-48) | 0.3 (0.19-0.44) | 0.01 (-0.51-0.54) |  |
| Bahamas | 1 (1-2) | 0.81 (0.59-1.02) | 3 (1-4) | 0.64 (0.31-1) | -0.56 (-0.7--0.41) |  |
| Bahrain | 0 (0-0) | 0.1 (0.07-0.14) | 0 (0-1) | 0.05 (0.03-0.07) | -3.12 (-3.31--2.93) |  |
| Bangladesh | 1 (0-3) | 0 (0-0.01) | 11 (0-23) | 0.01 (0-0.02) | 0 (0-0) |  |
| Barbados | 2 (1-2) | 0.63 (0.48-0.81) | 5 (4-7) | 1 (0.72-1.33) | 1.89 (1.67-2.11) |  |
| Belarus | 149 (93-204) | 1.14 (0.71-1.56) | 191 (113-284) | 1.19 (0.7-1.76) | -0.48 (-0.78--0.18) |  |
| Belgium | 370 (264-476) | 2.38 (1.73-3.06) | 300 (221-385) | 1.22 (0.9-1.55) | -1.87 (-2.07--1.66) |  |
| Belize | 0 (0-0) | 0.19 (0.14-0.25) | 1 (1-1) | 0.3 (0.21-0.38) | 1.76 (1.26-2.26) |  |
| Benin | 1 (1-2) | 0.07 (0.03-0.12) | 6 (3-10) | 0.12 (0.06-0.19) | 0 (0-0) |  |
| Bermuda | 1 (1-1) | 1.68 (1.26-2.14) | 1 (1-2) | 1.01 (0.72-1.36) | 0 (0-0) |  |
| Bhutan | 0 (0-1) | 0.11 (0.05-0.19) | 0 (0-0) | 0.02 (0-0.04) | 0 (0-0) |  |
| Bosnia and Herzegovina | 19 (11-28) | 0.46 (0.26-0.67) | 71 (49-99) | 1.13 (0.77-1.58) | 3.38 (3.09-3.68) |  |
| Botswana | 2 (1-2) | 0.27 (0.16-0.39) | 4 (3-7) | 0.28 (0.17-0.42) | 0 (0-0) |  |
| Brunei Darussalam | 0 (0-0) | 0.24 (0.16-0.34) | 0 (0-0) | 0.05 (0-0.09) | -1.6 (-3.52-0.35) |  |
| Bulgaria | 202 (140-260) | 1.66 (1.13-2.15) | 344 (258-456) | 2.42 (1.81-3.2) | 1.66 (1.51-1.82) |  |
| Burkina Faso | 9 (6-13) | 0.22 (0.14-0.32) | 26 (18-36) | 0.31 (0.2-0.43) | 0 (0-0) |  |
| Burundi | 13 (8-18) | 0.54 (0.33-0.77) | 17 (10-27) | 0.34 (0.2-0.54) | 0 (0-0) |  |
| Cabo Verde | 0 (0-0) | 0.13 (0.07-0.19) | 2 (1-3) | 0.4 (0.24-0.59) | 0 (0-0) |  |
| Cambodia | 6 (2-10) | 0.11 (0.05-0.19) | 83 (54-117) | 0.63 (0.42-0.88) | 5.36 (4.99-5.73) |  |
| Cameroon | 12 (7-17) | 0.27 (0.16-0.4) | 55 (34-81) | 0.45 (0.28-0.66) | 0 (0-0) |  |
| Canada | 353 (223-496) | 1.09 (0.69-1.52) | 671 (476-881) | 0.94 (0.68-1.22) | -0.05 (-0.25-0.14) |  |
| Central African Republic | 4 (2-6) | 0.32 (0.15-0.5) | 4 (2-8) | 0.18 (0.07-0.32) | 0 (0-0) |  |
| Chad | 1 (0-2) | 0.03 (0-0.09) | 10 (2-19) | 0.17 (0.04-0.33) | 0 (0-0) |  |
| Chile | 84 (64-106) | 0.86 (0.64-1.08) | 244 (184-309) | 0.95 (0.71-1.2) | 0.79 (0.54-1.04) |  |
| China | 5570 (3961-7269) | 0.65 (0.46-0.85) | 14937 (10533-20495) | 0.72 (0.51-0.99) | 0.48 (0.2-0.75) |  |
| Colombia | 53 (40-68) | 0.28 (0.21-0.36) | 139 (98-190) | 0.25 (0.18-0.35) | -0.62 (-0.94--0.3) |  |
| Congo | 3 (1-6) | 0.32 (0.13-0.54) | 14 (8-22) | 0.54 (0.31-0.82) | 0 (0-0) |  |
| Cook Islands | 0 (0-0) | 0.04 (0-0.12) | 0 (0-0) | 0.32 (0.22-0.43) | 0 (0-0) |  |
| Costa Rica | 7 (5-9) | 0.39 (0.29-0.5) | 31 (22-41) | 0.56 (0.41-0.74) | 1.22 (0.84-1.61) |  |
| Croatia | 107 (68-145) | 1.83 (1.14-2.48) | 174 (124-230) | 1.89 (1.37-2.48) | 0.08 (-0.16-0.31) |  |
| Cuba | 41 (30-54) | 0.4 (0.29-0.53) | 104 (76-140) | 0.54 (0.39-0.72) | 1.33 (1.16-1.51) |  |
| Cyprus | 9 (6-12) | 1.29 (0.86-1.75) | 17 (12-23) | 0.83 (0.6-1.09) | -0.93 (-1.23--0.64) |  |
| Czech | 466 (334-598) | 3.35 (2.4-4.28) | 460 (334-603) | 2.1 (1.55-2.74) | -1.93 (-2.14--1.72) |  |
| C么te d'Ivoire | 6 (3-10) | 0.17 (0.09-0.26) | 24 (14-38) | 0.23 (0.14-0.34) | 0 (0-0) |  |
| Democratic Korea | 73 (46-114) | 0.42 (0.27-0.64) | 123 (77-200) | 0.36 (0.23-0.59) | -0.19 (-0.38-0) |  |
| Denmark | 193 (137-248) | 2.35 (1.7-3.01) | 203 (150-265) | 1.61 (1.2-2.09) | -1.78 (-2.14--1.42) |  |
| Djibouti | 0 (0-0) | 0.09 (0.02-0.17) | 0 (0-0) | 0.02 (0-0.04) | 0 (0-0) |  |
| Dominica | 0 (0-0) | 0.49 (0.35-0.66) | 0 (0-1) | 0.59 (0.42-0.8) | 0.75 (0.6-0.91) |  |
| Dominican | 8 (6-12) | 0.21 (0.14-0.29) | 32 (22-45) | 0.31 (0.22-0.44) | 1.49 (1.2-1.78) |  |
| Ecuador | 6 (4-9) | 0.11 (0.07-0.15) | 36 (23-52) | 0.21 (0.14-0.31) | 3.09 (2.32-3.86) |  |
| Egypt | 2 (1-3) | 0.01 (0-0.01) | 9 (6-13) | 0.01 (0.01-0.02) | 3.79 (3.31-4.27) |  |
| El Salvador | 4 (3-5) | 0.11 (0.08-0.15) | 13 (9-17) | 0.21 (0.15-0.29) | 1.57 (1.26-1.88) |  |
| Equatorial Guinea | 0 (0-1) | 0.21 (0.08-0.37) | 3 (2-5) | 0.6 (0.33-0.93) | 0 (0-0) |  |
| Estonia | 19 (12-27) | 0.94 (0.57-1.3) | 32 (18-48) | 1.22 (0.73-1.76) | 0.81 (0.57-1.05) |  |
| Eswatini | 1 (1-2) | 0.43 (0.26-0.62) | 3 (2-5) | 0.56 (0.33-0.84) | 0 (0-0) |  |
| Ethiopia | 62 (19-118) | 0.3 (0.09-0.56) | 216 (118-349) | 0.51 (0.28-0.83) | 0 (0-0) |  |
| Federative Brazil | 299 (229-372) | 0.32 (0.25-0.41) | 1231 (960-1513) | 0.49 (0.38-0.6) | 1.32 (1-1.63) |  |
| Fiji | 0 (0-1) | 0.11 (0.06-0.17) | 1 (1-2) | 0.16 (0.09-0.24) | 1.42 (0.87-1.98) |  |
| Finland | 69 (47-90) | 0.98 (0.68-1.27) | 101 (74-130) | 0.79 (0.59-1.01) | -0.48 (-0.62--0.35) |  |
| French Republic | 2113 (1510-2706) | 2.5 (1.81-3.19) | 2180 (1625-2774) | 1.44 (1.1-1.82) | -1.72 (-1.78--1.66) |  |
| Gabonese Republic | 5 (3-9) | 0.98 (0.46-1.54) | 9 (6-13) | 0.89 (0.55-1.26) | 0 (0-0) |  |
| Gambia | 0 (0-0) | 0.03 (0.01-0.06) | 1 (0-1) | 0.08 (0.05-0.12) | 0 (0-0) |  |
| Georgia | 22 (13-36) | 0.35 (0.2-0.56) | 42 (27-58) | 0.73 (0.48-1.01) | 4.37 (3.65-5.09) |  |
| Germany | 3598 (2617-4584) | 2.79 (2.04-3.55) | 3111 (2388-3978) | 1.55 (1.21-1.96) | -2.11 (-2.2--2.01) |  |
| Ghana | 11 (6-16) | 0.18 (0.09-0.28) | 41 (25-61) | 0.26 (0.15-0.39) | 0 (0-0) |  |
| Grand Duchy of Luxembourg | 15 (11-19) | 2.69 (2-3.42) | 15 (11-19) | 1.35 (1.04-1.71) | -2.07 (-2.25--1.89) |  |
| Greenland | 1 (0-1) | 1.86 (1.02-2.83) | 1 (1-2) | 1.53 (0.91-2.19) | 0 (0-0) |  |
| Grenada | 0 (0-0) | 0.46 (0.33-0.62) | 1 (1-1) | 0.68 (0.5-0.91) | 0.93 (0.47-1.39) |  |
| Guam | 0 (0-0) | 0.17 (0-0.47) | 1 (0-1) | 0.33 (0.07-0.59) | 0 (0-0) |  |
| Guatemala | 4 (3-5) | 0.1 (0.07-0.13) | 16 (11-22) | 0.14 (0.1-0.19) | 1.14 (0.77-1.51) |  |
| Guinea | 1 (0-2) | 0.04 (0.01-0.07) | 4 (2-7) | 0.07 (0.03-0.13) | 0 (0-0) |  |
| Guinea-Bissau | 1 (1-1) | 0.25 (0.13-0.37) | 2 (1-3) | 0.26 (0.16-0.37) | 0 (0-0) |  |
| Guyana | 2 (2-3) | 0.57 (0.43-0.73) | 3 (2-5) | 0.5 (0.35-0.69) | -0.17 (-0.44-0.09) |  |
| Haiti | 17 (10-24) | 0.5 (0.32-0.73) | 35 (21-55) | 0.48 (0.28-0.75) | 0.11 (0.03-0.19) |  |
| Hashemite Jordan | 0 (0-0) | 0.02 (0.01-0.03) | 2 (1-2) | 0.02 (0.01-0.03) | 0 (0-0) |  |
| Hellenic Republic | 180 (129-235) | 1.19 (0.85-1.56) | 246 (180-312) | 0.98 (0.73-1.22) | -0.95 (-1.11--0.79) |  |
| Honduras | 2 (1-2) | 0.08 (0.06-0.11) | 8 (6-11) | 0.12 (0.09-0.18) | 1.44 (1.33-1.54) |  |
| Hungary | 377 (270-494) | 2.56 (1.85-3.37) | 430 (310-553) | 2.22 (1.61-2.86) | -0.62 (-0.81--0.43) |  |
| Iceland | 2 (1-3) | 0.62 (0.39-0.89) | 5 (3-6) | 0.81 (0.59-1.07) | 1.2 (0.9-1.49) |  |
| India | 307 (156-461) | 0.06 (0.03-0.09) | 1325 (894-1842) | 0.11 (0.07-0.15) | 0 (0-0) |  |
| Indonesia | 25 (8-43) | 0.02 (0.01-0.04) | 73 (23-138) | 0.03 (0.01-0.05) | 0.24 (-0.02-0.5) |  |
| Iran | 0 (0-0) | 0 (0-0) | 26 (17-35) | 0.03 (0.02-0.04) | 39.88 (32.44-47.73) |  |
| Iraq | 1 (1-1) | 0.01 (0.01-0.02) | 2 (1-3) | 0.01 (0.01-0.01) | -0.81 (-1.1--0.52) |  |
| Ireland | 80 (58-102) | 1.99 (1.46-2.52) | 91 (68-118) | 1.13 (0.85-1.47) | -1.99 (-2.15--1.83) |  |
| Italy | 1830 (1344-2298) | 2.05 (1.51-2.57) | 1919 (1428-2408) | 1.24 (0.95-1.54) | -1.77 (-1.9--1.64) |  |
| Jamaica | 4 (3-6) | 0.23 (0.16-0.32) | 13 (8-19) | 0.43 (0.27-0.63) | 2.3 (1.8-2.79) |  |
| Japan | 2264 (1721-2858) | 1.35 (1.02-1.7) | 3596 (2575-4725) | 0.99 (0.72-1.26) | -1.3 (-1.4--1.2) |  |
| Kazakhstan | 112 (73-154) | 0.84 (0.54-1.17) | 89 (62-123) | 0.48 (0.32-0.66) | -1.79 (-1.94--1.65) |  |
| Kenya | 16 (9-24) | 0.19 (0.12-0.28) | 64 (40-91) | 0.27 (0.17-0.38) | 0 (0-0) |  |
| Kiribati | 0 (0-0) | 0.08 (0.02-0.17) | 0 (0-0) | 0.04 (0-0.11) | -3.04 (-3.45--2.63) |  |
| Korea | 282 (209-371) | 0.92 (0.68-1.2) | 741 (541-998) | 0.8 (0.58-1.07) | -0.68 (-0.87--0.49) |  |
| Kyrgyz Republic | 10 (7-13) | 0.32 (0.22-0.42) | 11 (8-16) | 0.21 (0.14-0.3) | -1 (-1.27--0.73) |  |
| Lao People's Democratic Republic | 9 (4-16) | 0.4 (0.17-0.7) | 35 (22-52) | 0.7 (0.43-1.05) | 2.03 (1.64-2.43) |  |
| Latvia | 34 (21-47) | 0.95 (0.58-1.31) | 52 (31-76) | 1.36 (0.86-1.93) | 1.16 (0.97-1.35) |  |
| Lebanese Republic | 5 (3-7) | 0.24 (0.14-0.33) | 8 (5-12) | 0.13 (0.08-0.19) | 0 (0-0) |  |
| Lesotho | 2 (1-3) | 0.19 (0.1-0.3) | 5 (3-8) | 0.45 (0.25-0.7) | 0 (0-0) |  |
| Liberia | 2 (2-3) | 0.22 (0.14-0.31) | 4 (2-7) | 0.19 (0.09-0.32) | 0 (0-0) |  |
| Lithuania | 43 (26-61) | 0.96 (0.57-1.36) | 70 (40-106) | 1.25 (0.77-1.81) | 1.11 (0.7-1.53) |  |
| Madagascar | 8 (4-13) | 0.16 (0.07-0.25) | 13 (6-22) | 0.11 (0.05-0.18) | 0 (0-0) |  |
| Malawi | 3 (1-4) | 0.07 (0.04-0.1) | 9 (5-14) | 0.11 (0.07-0.17) | 0 (0-0) |  |
| Malaysia | 23 (15-33) | 0.25 (0.16-0.35) | 44 (25-66) | 0.15 (0.09-0.23) | -2.27 (-2.68--1.85) |  |
| Maldives | 0 (0-0) | 0.01 (0-0.05) | 0 (0-0) | 0.02 (0.01-0.04) | -2.51 (-4.46--0.51) |  |
| Mali | 3 (2-5) | 0.09 (0.05-0.14) | 8 (5-11) | 0.09 (0.06-0.14) | 0 (0-0) |  |
| Malta | 4 (3-5) | 0.94 (0.63-1.28) | 8 (6-10) | 0.79 (0.58-1.02) | -0.39 (-0.6--0.19) |  |
| Marshall Islands | 0 (0-0) | 0.12 (0.05-0.2) | 0 (0-0) | 0.17 (0.08-0.27) | 1.27 (1.15-1.4) |  |
| Mauritania | 0 (0-0) | 0 (0-0) | 0 (0-0) | 0 (0-0) | 0 (0-0) |  |
| Mauritius | 2 (1-2) | 0.24 (0.16-0.33) | 9 (6-12) | 0.46 (0.31-0.63) | 0 (0-0) |  |
| Micronesia | 0 (0-0) | 0.3 (0.16-0.46) | 0 (0-0) | 0.16 (0.08-0.27) | -2.38 (-2.55--2.21) |  |
| Moldova | 70 (45-95) | 1.56 (0.99-2.14) | 92 (58-127) | 1.52 (0.98-2.11) | 0.03 (-0.45-0.51) |  |
| Mongolia | 2 (1-3) | 0.17 (0.09-0.26) | 10 (7-15) | 0.4 (0.27-0.59) | 4.13 (3.73-4.53) |  |
| Montenegro | 6 (4-9) | 1 (0.64-1.43) | 12 (9-17) | 1.23 (0.88-1.69) | 0.84 (0.73-0.96) |  |
| Morocco | 1 (1-2) | 0.01 (0-0.01) | 3 (2-5) | 0.01 (0-0.01) | 0 (0-0) |  |
| Mozambique | 1 (0-2) | 0.01 (0-0.03) | 6 (3-11) | 0.06 (0.03-0.1) | 0 (0-0) |  |
| Namibia | 1 (1-2) | 0.2 (0.09-0.33) | 6 (4-8) | 0.41 (0.26-0.58) | 0 (0-0) |  |
| Nauru | 0 (0-0) | 0.53 (0.2-1) | 0 (0-0) | 0.55 (0.3-0.83) | 0 (0-0) |  |
| Nepal | 1 (0-1) | 0 (0-0.01) | 20 (8-34) | 0.08 (0.03-0.14) | 0 (0-0) |  |
| Netherlands | 418 (300-532) | 2.08 (1.5-2.65) | 629 (480-808) | 1.73 (1.32-2.21) | -0.56 (-0.77--0.36) |  |
| New Zealand | 109 (69-148) | 2.81 (1.82-3.84) | 138 (100-180) | 1.61 (1.16-2.09) | -1.8 (-1.9--1.7) |  |
| Nicaragua | 2 (1-2) | 0.1 (0.07-0.14) | 9 (6-12) | 0.17 (0.12-0.24) | 2.4 (2-2.8) |  |
| Niger | 0 (0-0) | 0 (0-0.01) | 1 (0-2) | 0.01 (0-0.03) | 0 (0-0) |  |
| Nigeria | 88 (55-125) | 0.21 (0.13-0.29) | 236 (155-332) | 0.28 (0.19-0.39) | 0 (0-0) |  |
| Niue | 0 (0-0) | 0.26 (0.04-0.46) | 0 (0-0) | 0.39 (0.15-0.56) | 0 (0-0) |  |
| North Macedonia | 23 (16-31) | 1.26 (0.84-1.71) | 40 (29-54) | 1.22 (0.86-1.64) | -0.31 (-0.49--0.13) |  |
| Northern Mariana Islands | 0 (0-0) | 0.23 (0-0.54) | 0 (0-0) | 0.35 (0.04-0.67) | 0 (0-0) |  |
| Norway | 75 (49-105) | 1.13 (0.75-1.55) | 115 (83-153) | 1.11 (0.8-1.45) | 0.07 (-0.2-0.33) |  |
| Pakistan | 7 (1-13) | 0.01 (0-0.02) | 38 (18-63) | 0.03 (0.01-0.05) | 0 (0-0) |  |
| Palau | 0 (0-0) | 0.11 (0.01-0.27) | 0 (0-0) | 0.15 (0.03-0.32) | 0 (0-0) |  |
| Palestine | 1 (1-1) | 0.1 (0.06-0.15) | 2 (1-3) | 0.08 (0.05-0.11) | 0 (0-0) |  |
| Panama | 5 (4-6) | 0.33 (0.24-0.41) | 20 (13-26) | 0.44 (0.3-0.59) | 1.43 (1.22-1.64) |  |
| Papua New Guinea | 1 (0-1) | 0.03 (0.02-0.05) | 1 (1-2) | 0.02 (0.01-0.04) | -0.5 (-1.11-0.12) |  |
| Paraguay | 9 (7-12) | 0.41 (0.3-0.53) | 36 (25-50) | 0.62 (0.43-0.85) | 1.32 (1.18-1.47) |  |
| People's Democratic Algeria | 1 (1-2) | 0.01 (0.01-0.02) | 6 (4-9) | 0.02 (0.01-0.03) | 1.84 (1.66-2.01) |  |
| Peru | 41 (25-59) | 0.33 (0.2-0.48) | 139 (89-202) | 0.41 (0.26-0.59) | 0.99 (0.63-1.35) |  |
| Philippines | 171 (120-221) | 0.52 (0.37-0.68) | 690 (513-907) | 0.78 (0.58-1.03) | 1.58 (1.5-1.66) |  |
| Plurinational State of Bolivia | 13 (7-19) | 0.39 (0.23-0.6) | 41 (27-63) | 0.46 (0.3-0.68) | 0.88 (0.62-1.14) |  |
| Poland | 575 (415-728) | 1.31 (0.94-1.66) | 1362 (1004-1710) | 1.89 (1.41-2.36) | 1.27 (1.11-1.43) |  |
| Portuguese Republic | 277 (203-361) | 2.03 (1.47-2.62) | 398 (303-514) | 1.58 (1.22-2.03) | -0.79 (-0.99--0.59) |  |
| Principality of Andorra | 2 (1-2) | 2.69 (1.67-4.08) | 2 (2-3) | 1.56 (0.99-2.2) | -1.54 (-1.77--1.31) |  |
| Principality of Monaco | 1 (0-2) | 1.26 (0.02-2.72) | 1 (0-3) | 1.49 (0.06-3.06) | 0 (0-0) |  |
| Puerto Rico | 18 (13-24) | 0.51 (0.37-0.67) | 33 (24-43) | 0.52 (0.38-0.68) | 0 (0-0) |  |
| Romania | 273 (192-365) | 0.97 (0.67-1.3) | 682 (488-914) | 1.86 (1.35-2.46) | 1.98 (1.75-2.2) |  |
| Russian Federation | 1840 (1269-2369) | 1 (0.68-1.28) | 2391 (1583-3281) | 1.01 (0.67-1.38) | -0.31 (-0.71-0.09) |  |
| Rwanda | 19 (11-27) | 0.64 (0.38-0.92) | 28 (16-42) | 0.44 (0.26-0.64) | 0 (0-0) |  |
| Saint Kitts and Nevis | 0 (0-0) | 0.45 (0-0.64) | 0 (0-1) | 0.35 (0-0.87) | 0 (0-0) |  |
| Saint Lucia | 1 (0-1) | 0.61 (0.46-0.76) | 1 (1-2) | 0.56 (0.4-0.73) | -0.45 (-0.63--0.27) |  |
| Saint Vincent and the Grenadines | 0 (0-0) | 0.33 (0.21-0.45) | 1 (1-1) | 0.72 (0.54-0.91) | 2.58 (2.31-2.85) |  |
| Samoa | 0 (0-0) | 0.14 (0.08-0.2) | 0 (0-0) | 0.09 (0.05-0.14) | -1.58 (-2.01--1.15) |  |
| San Marino | 1 (0-1) | 1.81 (0-3.07) | 1 (0-1) | 0.92 (0-1.7) | 0 (0-0) |  |
| Sao Tome and Principe | 0 (0-0) | 0.39 (0.24-0.54) | 1 (0-1) | 0.67 (0.46-0.9) | 0 (0-0) |  |
| Saudi Arabia | 1 (0-1) | 0.01 (0-0.02) | 2 (0-4) | 0.01 (0-0.02) | 0 (0-0) |  |
| Senegal | 1 (0-2) | 0.04 (0.01-0.07) | 2 (1-4) | 0.03 (0.01-0.05) | 0 (0-0) |  |
| Serbia | 156 (95-225) | 1.45 (0.85-2.1) | 247 (174-335) | 1.48 (1.05-2.01) | 0.18 (0.07-0.3) |  |
| Seychelles | 0 (0-0) | 0.42 (0.29-0.57) | 1 (0-1) | 0.56 (0.38-0.75) | 0 (0-0) |  |
| Sierra Leone | 4 (2-5) | 0.18 (0.12-0.26) | 6 (4-8) | 0.15 (0.1-0.22) | 0 (0-0) |  |
| Singapore | 7 (5-10) | 0.3 (0.19-0.42) | 14 (9-20) | 0.16 (0.11-0.23) | -2.77 (-3.22--2.33) |  |
| Slovak Republic | 160 (114-210) | 2.65 (1.89-3.48) | 215 (160-282) | 2.23 (1.65-2.92) | -0.65 (-0.73--0.57) |  |
| Slovenia | 43 (28-58) | 1.71 (1.12-2.33) | 40 (16-67) | 0.87 (0.37-1.44) | -2.54 (-2.68--2.39) |  |
| Socialist Viet Nam | 8 (1-16) | 0.02 (0-0.04) | 878 (601-1168) | 0.87 (0.6-1.15) | 13.48 (11.98-14.99) |  |
| Solomon Islands | 0 (0-0) | 0.02 (0.01-0.05) | 0 (0-0) | 0.05 (0.02-0.09) | 3.74 (2.95-4.54) |  |
| Somalia | 0 (0-0) | 0 (0-0) | 0 (0-0) | 0 (0-0) | 0 (0-0) |  |
| South Africa | 108 (78-149) | 0.51 (0.37-0.71) | 273 (199-357) | 0.59 (0.42-0.76) | 0 (0-0) |  |
| South Sudan | 1 (0-1) | 0.02 (0-0.05) | 1 (0-2) | 0.02 (0-0.04) | 0 (0-0) |  |
| Spain | 893 (654-1153) | 1.66 (1.23-2.15) | 1308 (958-1676) | 1.3 (0.97-1.67) | -0.91 (-1.05--0.77) |  |
| Sri Lanka | 9 (6-12) | 0.08 (0.06-0.11) | 30 (17-45) | 0.11 (0.06-0.16) | 1.04 (0.73-1.35) |  |
| State of Eritrea | 1 (0-3) | 0.11 (0.03-0.19) | 3 (1-6) | 0.09 (0.03-0.17) | 0 (0-0) |  |
| State of Israel | 11 (5-19) | 0.23 (0.1-0.4) | 38 (24-55) | 0.31 (0.19-0.44) | 1.06 (0.71-1.41) |  |
| State of Kuwait | 0 (0-0) | 0 (0-0) | 0 (0-0) | 0 (0-0.01) | 0 (0-0) |  |
| State of Libya | 0 (0-0) | 0 (0-0) | 2 (1-3) | 0.03 (0.02-0.05) | 0 (0-0) |  |
| State of Qatar | 0 (0-0) | 0.04 (0.02-0.06) | 0 (0-1) | 0.03 (0.02-0.04) | 0 (0-0) |  |
| Sudan | 7 (4-12) | 0.08 (0.05-0.12) | 0 (0-0) | 0 (0-0) | 0 (0-0) |  |
| Sultanate of Oman | 0 (0-0) | 0.01 (0-0.01) | 0 (0-0) | 0.01 (0-0.01) | 0 (0-0) |  |
| Suriname | 1 (1-2) | 0.47 (0.34-0.62) | 3 (2-4) | 0.46 (0.31-0.67) | 0.31 (0.07-0.56) |  |
| Sweden | 205 (134-273) | 1.36 (0.93-1.78) | 233 (161-314) | 1.02 (0.72-1.35) | -0.68 (-0.82--0.54) |  |
| Swiss Confederation | 161 (115-205) | 1.52 (1.11-1.93) | 174 (128-227) | 0.9 (0.67-1.17) | -1.74 (-1.93--1.54) |  |
| Syrian Arab Republic | 3 (2-4) | 0.04 (0.03-0.07) | 3 (1-5) | 0.02 (0.01-0.04) | 0 (0-0) |  |
| Taiwan (Province of China) | 143 (113-177) | 0.87 (0.68-1.07) | 317 (239-404) | 0.76 (0.57-0.96) | -1.23 (-1.55--0.9) |  |
| Tajikistan | 4 (3-6) | 0.13 (0.09-0.18) | 5 (3-8) | 0.06 (0.04-0.1) | -2.72 (-3.24--2.19) |  |
| Thailand | 174 (124-231) | 0.47 (0.33-0.63) | 680 (443-968) | 0.64 (0.42-0.91) | 0.76 (0.63-0.9) |  |
| the Congo | 26 (9-44) | 0.17 (0.06-0.29) | 55 (21-104) | 0.15 (0.06-0.28) | 0 (0-0) |  |
| Timor-Leste | 0 (0-0) | 0.09 (0.05-0.16) | 2 (1-3) | 0.22 (0.13-0.31) | 2.53 (2.24-2.81) |  |
| Togolese Republic | 1 (1-2) | 0.12 (0.07-0.18) | 5 (3-8) | 0.13 (0.07-0.22) | 0 (0-0) |  |
| Tokelau | 0 (0-0) | 0.14 (0.05-0.25) | 0 (0-0) | 0.21 (0.09-0.35) | 0 (0-0) |  |
| Tonga | 0 (0-0) | 0.04 (0.02-0.06) | 0 (0-0) | 0.03 (0.01-0.06) | -0.95 (-1.85--0.05) |  |
| Trinidad and Tobago | 4 (3-6) | 0.53 (0.38-0.69) | 12 (8-17) | 0.62 (0.43-0.89) | 1.05 (0.82-1.29) |  |
| Tunisia | 2 (2-3) | 0.05 (0.03-0.07) | 8 (5-13) | 0.06 (0.04-0.09) | 0 (0-0) |  |
| Turkey | 68 (47-95) | 0.18 (0.12-0.25) | 148 (100-207) | 0.15 (0.1-0.22) | 0 (0-0) |  |
| Turkmenistan | 3 (1-4) | 0.13 (0.07-0.21) | 9 (6-13) | 0.21 (0.13-0.31) | 1.88 (1.57-2.2) |  |
| Tuvalu | 0 (0-0) | 0.1 (0.04-0.18) | 0 (0-0) | 0.13 (0.06-0.23) | 0 (0-0) |  |
| Uganda | 25 (14-37) | 0.38 (0.22-0.55) | 82 (53-119) | 0.52 (0.34-0.75) | 0 (0-0) |  |
| Ukraine | 846 (532-1119) | 1.18 (0.75-1.56) | 699 (361-1084) | 0.92 (0.49-1.41) | -0.59 (-0.98--0.2) |  |
| Union of Myanmar | 7 (3-16) | 0.03 (0.01-0.06) | 143 (93-209) | 0.28 (0.18-0.41) | 9.01 (8.49-9.53) |  |
| Union of the Comoros | 0 (0-0) | 0.01 (0-0.03) | 0 (0-0) | 0.04 (0.01-0.08) | 0 (0-0) |  |
| United Arab Emirates | 2 (1-3) | 0.26 (0.13-0.44) | 5 (3-9) | 0.11 (0.06-0.2) | 0 (0-0) |  |
| United Great Britain and Northern Ireland | 1612 (1096-2077) | 1.79 (1.25-2.29) | 1694 (1272-2113) | 1.27 (0.95-1.56) | -1.24 (-1.36--1.13) |  |
| United Mexican States | 94 (73-118) | 0.21 (0.16-0.26) | 495 (366-640) | 0.38 (0.28-0.49) | 1.9 (1.61-2.19) |  |
| United States of America | 3183 (2035-4450) | 1.01 (0.67-1.41) | 4687 (3584-5854) | 0.84 (0.64-1.04) | -0.53 (-0.59--0.46) |  |
| United States Virgin Islands | 1 (0-1) | 0.68 (0-1.54) | 1 (0-2) | 0.72 (0.03-1.36) | 0 (0-0) |  |
| United Tanzania | 37 (21-54) | 0.34 (0.2-0.49) | 106 (71-151) | 0.43 (0.29-0.6) | 0 (0-0) |  |
| Uruguay | 65 (50-83) | 1.66 (1.27-2.13) | 94 (70-120) | 1.66 (1.24-2.1) | -0.05 (-0.17-0.07) |  |
| Uzbekistan | 17 (11-26) | 0.14 (0.08-0.2) | 46 (31-61) | 0.15 (0.1-0.21) | 0.23 (-0.31-0.77) |  |
| Vanuatu | 0 (0-0) | 0.12 (0.06-0.19) | 0 (0-0) | 0.15 (0.09-0.22) | 1 (-0.25-2.25) |  |
| Venezuela | 41 (32-51) | 0.4 (0.31-0.5) | 95 (59-136) | 0.31 (0.2-0.45) | -0.69 (-0.98--0.4) |  |
| Yemen | 2 (1-4) | 0.04 (0.02-0.07) | 2 (1-3) | 0.01 (0.01-0.02) | 0 (0-0) |  |
| Zambia | 10 (6-15) | 0.36 (0.2-0.53) | 45 (23-100) | 0.6 (0.33-1.24) | 0 (0-0) |  |
| Zimbabwe | 15 (9-22) | 0.38 (0.21-0.54) | 31 (18-46) | 0.43 (0.26-0.63) | 0 (0-0) |  |
