# Supplementary material for: The global, regional, and national alcohol-related colorectal cancer burden and forecasted trends: results from the global burden of disease study 2021
Source: Front Nutr. 2024 Dec 24;11:1520852. doi: 10.3389/fnut.2024.1520852 (PMC11704491; doi:10.3389/fnut.2024.1520852)
Supplement: Supplementary file 14 [file Table_4.docx]

**Supplementary Table 4.** The number of DALYs cases and the age-standardized DALYs rate of Alcohol-related colorectal cancer in 1990 and 2021, and its trends from 1990 to 2021 globally.

|  | Number of DALYs cases (95% UI) in 1990 | The age-standardized DALYs rate/100000 (95% UI) in 1990 | Number of DALYs cases (95% UI) in 2021 | The age-standardized DALYs rate/100000 (95% UI) in 2021 | EAPC (95% CI) |  |
| --- | --- | --- | --- | --- | --- | --- |
| Global | 892190 (707015-1089013) | 21.74 (16.95-26.65) | 1425324 (1122455-1769389) | 16.44 (12.95-20.43) | -0.94 (-0.98--0.91) |  |
| Sex |  |  |  |  |  |  |
| Female | 206427 (159547-261906) | 9.66 (7.4-12.26) | 227476 (174133-288659) | 4.99 (3.83-6.34) | -2.21 (-2.27--2.16) |  |
| Male | 685763 (536295-833960) | 35.63 (27.62-43.63) | 1197848 (932790-1488851) | 29.14 (22.67-36.29) | -0.68 (-0.72--0.64) |  |
| Age |  |  |  |  |  |  |
| 15-19 years | 1807 (1267-2387) | 0.35 (0.24-0.46) | 1367 (997-1821) | 0.22 (0.16-0.29) | -1.51 (-1.62--1.4) |  |
| 20-24 years | 7732 (5533-9917) | 1.57 (1.12-2.02) | 6544 (4944-8559) | 1.1 (0.83-1.43) | -1.12 (-1.32--0.92) |  |
| 25-29 years | 12859 (9627-16540) | 2.91 (2.17-3.74) | 13772 (10586-17774) | 2.34 (1.8-3.02) | -0.61 (-0.81--0.4) |  |
| 30-34 years | 22964 (17246-28875) | 5.96 (4.47-7.49) | 30940 (23723-40323) | 5.12 (3.92-6.67) | -0.63 (-0.83--0.42) |  |
| 35-39 years | 37541 (28957-47025) | 10.66 (8.22-13.35) | 46352 (35514-59252) | 8.26 (6.33-10.56) | -0.97 (-1.14--0.81) |  |
| 40-44 years | 50042 (38828-61723) | 17.47 (13.55-21.55) | 65347 (50115-84138) | 13.06 (10.02-16.82) | -1 (-1.14--0.86) |  |
| 45-49 years | 66052 (51472-80864) | 28.45 (22.17-34.83) | 101026 (78033-128845) | 21.34 (16.48-27.21) | -0.91 (-1.02--0.81) |  |
| 50-54 years | 95243 (76255-115514) | 44.81 (35.87-54.34) | 147402 (113787-190193) | 33.13 (25.57-42.75) | -1.01 (-1.06--0.95) |  |
| 55-59 years | 116699 (93116-140901) | 63.01 (50.28-76.08) | 184856 (143906-230331) | 46.71 (36.36-58.2) | -0.97 (-1.04--0.9) |  |
| 60-64 years | 134823 (108030-164383) | 83.94 (67.26-102.35) | 197282 (154431-244255) | 61.64 (48.25-76.32) | -0.96 (-1.04--0.88) |  |
| 65-69 years | 121325 (92608-150080) | 98.15 (74.92-121.42) | 199088 (158106-247424) | 72.17 (57.32-89.7) | -1.1 (-1.16--1.04) |  |
| 70-74 years | 85667 (63376-108122) | 101.19 (74.86-127.71) | 173792 (138535-218508) | 84.43 (67.3-106.15) | -0.96 (-1.09--0.82) |  |
| 75-79 years | 74958 (52331-96344) | 121.77 (85.02-156.52) | 115348 (86950-145064) | 87.46 (65.93-109.99) | -0.88 (-1.01--0.76) |  |
| 80-84 years | 39837 (25384-52383) | 112.61 (71.76-148.08) | 71137 (52409-91536) | 81.22 (59.84-104.51) | -0.94 (-1.07--0.81) |  |
| 85-89 years | 17952 (10691-23981) | 118.8 (70.75-158.7) | 44159 (31184-57032) | 96.58 (68.2-124.74) | -0.63 (-0.74--0.53) |  |
| 90-94 years | 5506 (2992-7657) | 128.49 (69.83-178.68) | 20445 (13495-26726) | 114.29 (75.44-149.4) | -0.43 (-0.55--0.32) |  |
| 95+ years | 1181 (571-1714) | 116.03 (56.12-168.4) | 6469 (4147-8734) | 118.68 (76.1-160.25) | -0.11 (-0.22-0.01) |  |
| SDI region |  |  |  |  |  |  |
| High-middle SDI | 306775 (242262-376953) | 29.81 (23.53-36.68) | 461951 (363004-576454) | 23.92 (18.73-29.87) | -0.8 (-0.87--0.73) |  |
| High SDI | 429326 (330053-533046) | 39.98 (30.83-49.59) | 513549 (408432-624443) | 27.17 (21.81-32.97) | -1.32 (-1.35--1.29) |  |
| Low-middle SDI | 17285 (12030-22949) | 2.41 (1.68-3.2) | 63880 (47792-81466) | 3.97 (2.97-5.05) | 1.91 (1.79-2.02) |  |
| Low SDI | 9414 (5327-13051) | 3.62 (2.13-5) | 26010 (17945-34955) | 4.44 (3.12-5.88) | 0.6 (0.28-0.92) |  |
| Middle SDI | 127990 (93754-162875) | 10.45 (7.7-13.28) | 357898 (273863-467239) | 12.71 (9.72-16.57) | 0.77 (0.57-0.97) |  |
| GBD region |  |  |  |  |  |  |
| Advanced Health System | 617963 (473356-763950) | 38.6 (29.85-47.74) | 720062 (565673-876030) | 27.73 (22.11-33.77) | -1.17 (-1.27--1.08) |  |
| Africa | 16079 (10038-21213) | 5.02 (3.17-6.61) | 45586 (32403-61718) | 6.06 (4.35-8.05) | 0.59 (0.53-0.65) |  |
| African Region | 15624 (9642-20698) | 6.22 (3.91-8.21) | 44825 (31790-60909) | 7.63 (5.47-10.14) | 0.65 (0.58-0.71) |  |
| America | 132295 (100436-170371) | 21.46 (16.21-27.67) | 236751 (187524-289385) | 18.37 (14.64-22.47) | -0.34 (-0.39--0.28) |  |
| Andean Latin America | 1862 (1209-2581) | 8 (5.17-11.08) | 6277 (4257-8583) | 10.12 (6.87-13.8) | 1.18 (0.97-1.38) |  |
| Asia | 303699 (227196-376379) | 13.35 (10.05-16.51) | 674996 (512198-874910) | 13.01 (9.92-16.86) | -0.07 (-0.25-0.1) |  |
| Australasia | 12852 (8689-17165) | 56.15 (38.48-74.44) | 15957 (12279-20234) | 32.59 (25.45-41.36) | -1.88 (-1.94--1.81) |  |
| Basic Health System | 245378 (183341-308593) | 14.46 (10.78-18.21) | 608287 (461510-793082) | 15.77 (12.02-20.58) | 0.43 (0.22-0.63) |  |
| Caribbean | 3152 (2379-3940) | 11.48 (8.7-14.39) | 7176 (5437-9153) | 13.46 (10.2-17.17) | 0.79 (0.71-0.87) |  |
| Central Africa | 1934 (1044-2784) | 6.07 (3.38-8.69) | 5213 (3110-7763) | 6.62 (4.11-9.74) | 0.65 (0.09-1.21) |  |
| Central Asia | 6749 (4907-8731) | 12.87 (9.3-16.72) | 8237 (6168-10760) | 8.94 (6.66-11.72) | -0.85 (-1.04--0.66) |  |
| Central Europe | 64150 (49306-80207) | 42.47 (32.7-53.13) | 96851 (75901-119489) | 46.57 (36.49-57.33) | 0.26 (0.15-0.37) |  |
| Central Latin America | 6999 (5565-8652) | 6.96 (5.55-8.64) | 26150 (20334-32970) | 9.96 (7.76-12.57) | 1.06 (0.89-1.22) |  |
| Central Sub-Saharan Africa | 1438 (620-2229) | 5.56 (2.52-8.6) | 4294 (2369-6599) | 6.39 (3.5-9.81) | 1.12 (0.48-1.77) |  |
| Commonwealth High Income | 60123 (44272-76544) | 42.1 (31.52-53.37) | 68864 (53499-85292) | 28.04 (21.92-34.43) | -1.35 (-1.43--1.27) |  |
| Commonwealth Low Income | 3160 (1786-4357) | 3.22 (1.93-4.42) | 9589 (6625-13465) | 3.67 (2.57-5.11) | 0.16 (0.07-0.25) |  |
| Commonwealth Middle Income | 18879 (11751-25565) | 2.59 (1.64-3.48) | 65043 (45962-86765) | 3.74 (2.65-4.93) | 1.27 (1.15-1.38) |  |
| East Asia | 188826 (132672-244799) | 18.9 (13.38-24.54) | 434302 (311168-591949) | 20.23 (14.54-27.64) | 0.31 (0.02-0.61) |  |
| East Asia & Pacific - WB | 287062 (213943-358137) | 19.33 (14.44-24.06) | 622948 (472131-816583) | 19.13 (14.55-25.12) | -0.04 (-0.22-0.15) |  |
| Eastern Africa | 5572 (2891-7970) | 6.88 (3.77-9.82) | 15781 (10572-21754) | 8.48 (5.73-11.69) | 0.48 (0.22-0.75) |  |
| Eastern Europe | 89450 (64655-112104) | 31.76 (23.21-39.73) | 96163 (69098-126468) | 28.85 (21.3-37.71) | -0.54 (-0.88--0.19) |  |
| Eastern Mediterranean Region | 1175 (797-1620) | 0.54 (0.37-0.75) | 3848 (2658-5324) | 0.68 (0.47-0.93) | 1.17 (0.94-1.4) |  |
| Eastern Sub-Saharan Africa | 6073 (3040-8640) | 7 (3.71-9.88) | 18053 (11994-25605) | 9.1 (6.11-12.89) | 0.71 (0.44-0.99) |  |
| Europe | 438112 (327784-542540) | 43.14 (32.64-53.36) | 464990 (357373-573202) | 31.56 (24.54-38.86) | -1.12 (-1.26--0.98) |  |
| Europe & Central Asia - WB | 442354 (331102-547455) | 42.29 (31.96-52.29) | 469103 (360846-578239) | 30.58 (23.76-37.69) | -1.16 (-1.3--1.02) |  |
| European Region | 443311 (331766-548647) | 42.1 (31.81-52.06) | 471109 (362397-580726) | 30.39 (23.62-37.46) | -1.16 (-1.3--1.02) |  |
| High-income Asia Pacific | 70740 (54309-87809) | 34.4 (26.41-42.71) | 91502 (68605-115743) | 23.67 (17.92-29.85) | -1.48 (-1.57--1.4) |  |
| High-income North America | 92281 (65584-123202) | 27.91 (20.19-36.83) | 136304 (105529-167428) | 23.74 (18.69-29.13) | -0.36 (-0.44--0.28) |  |
| Latin America & Caribbean - WB | 40607 (32419-49610) | 14.03 (11.11-17.2) | 101384 (80138-123791) | 14.12 (11.16-17.25) | 0.15 (0.09-0.2) |  |
| Limited Health System | 25509 (15771-34348) | 2.74 (1.75-3.7) | 90397 (65814-117113) | 4.06 (2.96-5.19) | 1.42 (1.23-1.61) |  |
| Middle East & North Africa - WB | 1133 (810-1556) | 0.81 (0.56-1.13) | 3773 (2828-4912) | 0.98 (0.73-1.27) | 1.1 (0.88-1.33) |  |
| Minimal Health System | 1939 (1022-2793) | 2.9 (1.56-4.15) | 4542 (2654-7135) | 3.12 (1.87-4.78) | 0.37 (-0.15-0.9) |  |
| North Africa and Middle East | 3364 (2373-4542) | 1.63 (1.15-2.19) | 7148 (5145-9405) | 1.37 (0.97-1.8) | -0.7 (-0.88--0.52) |  |
| North America | 92284 (65589-123205) | 27.91 (20.19-36.83) | 136304 (105532-167418) | 23.74 (18.69-29.12) | -0.36 (-0.44--0.28) |  |
| Northern Africa | 257 (185-342) | 0.33 (0.24-0.44) | 951 (661-1328) | 0.52 (0.36-0.73) | 1.81 (1.64-1.97) |  |
| Oceania | 74 (45-108) | 1.86 (1.11-2.7) | 171 (110-234) | 1.79 (1.14-2.43) | 0.15 (-0.13-0.44) |  |
| Region of the Americas | 132295 (100436-170371) | 21.46 (16.21-27.67) | 236751 (187524-289385) | 18.37 (14.64-22.47) | -0.34 (-0.39--0.28) |  |
| South-East Asia Region | 20049 (13360-26654) | 2.35 (1.57-3.12) | 74224 (54666-94915) | 3.73 (2.76-4.77) | 1.55 (1.44-1.66) |  |
| South Asia | 10635 (5392-15754) | 1.52 (0.77-2.27) | 43282 (29478-59608) | 2.64 (1.79-3.65) | 2.01 (1.77-2.25) |  |
| South Asia - WB | 10929 (5679-16124) | 1.52 (0.78-2.26) | 44166 (30312-60639) | 2.63 (1.79-3.62) | 2 (1.78-2.22) |  |
| Southeast Asia | 14891 (10861-19343) | 4.78 (3.48-6.21) | 81917 (62156-103056) | 11.28 (8.59-14.22) | 3.04 (2.93-3.15) |  |
| Southern Africa | 4710 (3146-6240) | 9.32 (6.32-12.32) | 13361 (9320-18740) | 12.05 (8.53-16.61) | 0.84 (0.65-1.02) |  |
| Southern Latin America | 18947 (15066-23411) | 40.46 (32.1-50.01) | 24808 (18966-31302) | 29.43 (22.55-37.17) | -0.62 (-0.74--0.49) |  |
| Southern Sub-Saharan Africa | 4028 (2763-5320) | 12.97 (9.11-17.24) | 9797 (6999-12846) | 15.06 (10.88-19.42) | 0.42 (0.21-0.62) |  |
| Sub-Saharan Africa - WB | 15818 (9788-20898) | 6.31 (3.96-8.3) | 44630 (31601-60662) | 7.74 (5.55-10.29) | 0.65 (0.58-0.71) |  |
| Tropical Latin America | 9784 (7618-12081) | 9.23 (7.12-11.41) | 37251 (29714-45506) | 14.16 (11.29-17.28) | 1.33 (1.03-1.63) |  |
| Western Africa | 3606 (2355-4914) | 4.23 (2.79-5.75) | 10279 (7123-13891) | 5.31 (3.75-7.01) | 0.79 (0.7-0.88) |  |
| Western Europe | 281913 (212980-348101) | 51.23 (39.45-63.17) | 267444 (208787-329828) | 31.67 (24.93-38.94) | -1.65 (-1.73--1.56) |  |
| Western Pacific Region | 273116 (202575-342268) | 21.58 (16.06-26.99) | 582977 (438138-773319) | 21.23 (15.96-28.26) | -0.05 (-0.25-0.15) |  |
| Western Sub-Saharan Africa | 3981 (2686-5454) | 4.21 (2.84-5.76) | 12240 (8579-16237) | 5.61 (3.98-7.31) | 1.04 (0.95-1.13) |  |
| World Bank High Income | 521729 (400764-645675) | 41.78 (32.51-51.69) | 611781 (483624-745817) | 28.77 (22.85-35) | -1.28 (-1.34--1.23) |  |
| World Bank Low Income | 8961 (5409-12655) | 5.19 (3.19-7.34) | 21008 (13773-29603) | 5.48 (3.67-7.6) | 0.07 (-0.27-0.41) |  |
| World Bank Lower Middle Income | 52758 (38645-67398) | 4.43 (3.21-5.67) | 149139 (111042-188308) | 5.33 (3.99-6.71) | 0.76 (0.68-0.83) |  |
| World Bank Upper Middle Income | 307341 (238713-382207) | 18.34 (14.16-22.88) | 641358 (491871-833153) | 18.25 (13.99-23.73) | -0.04 (-0.16-0.07) |  |
| Country |  |  |  |  |  |  |
| Afghanistan | 0 (0-0) | 0 (0-0) | 17 (6-35) | 0.1 (0.03-0.21) | 0 (0-0) |  |
| Albania | 77 (42-122) | 3.48 (1.85-5.52) | 325 (210-496) | 7.58 (4.91-11.58) | 2.88 (2.11-3.65) |  |
| American Samoa | 0 (0-1) | 1.46 (0.17-4.28) | 1 (0-2) | 1.22 (0.25-3.05) | 0 (0-0) |  |
| Angola | 265 (112-433) | 5.46 (2.35-8.89) | 1578 (922-2340) | 10.6 (6.32-15.35) | 0 (0-0) |  |
| Antigua and Barbuda | 2 (1-4) | 4.55 (1.91-7.49) | 13 (10-17) | 12.03 (8.83-15.34) | 3.21 (2.69-3.73) |  |
| Argentine Republic | 15140 (11971-18791) | 46.45 (36.75-57.64) | 16835 (12865-21656) | 31.09 (23.82-40.01) | -0.79 (-0.98--0.6) |  |
| Armenia | 313 (193-436) | 10.06 (6.12-14.16) | 464 (321-624) | 11.14 (7.83-14.88) | 0.64 (0.31-0.97) |  |
| Australia | 10093 (6871-13536) | 52.83 (36.41-70.43) | 12910 (9806-16460) | 31.51 (24.28-40) | -1.81 (-1.88--1.73) |  |
| Austria | 6382 (4837-8016) | 57.34 (44.36-71.86) | 4159 (3208-5202) | 24.94 (19.31-31.3) | -2.66 (-2.73--2.59) |  |
| Azerbaijan | 698 (466-959) | 12 (7.91-16.57) | 1039 (665-1491) | 8.76 (5.6-12.8) | -0.49 (-1.02-0.05) |  |
| Bahamas | 45 (33-57) | 24.94 (18.35-31.43) | 83 (43-128) | 18.87 (9.73-29.13) | -0.67 (-0.82--0.52) |  |
| Bahrain | 7 (5-9) | 2.65 (1.88-3.66) | 17 (12-26) | 1.27 (0.85-1.93) | -3.01 (-3.21--2.81) |  |
| Bangladesh | 36 (0-116) | 0.06 (0-0.21) | 367 (17-759) | 0.24 (0.01-0.5) | 0 (0-0) |  |
| Barbados | 47 (36-60) | 17.99 (13.82-22.64) | 127 (90-170) | 26.35 (18.74-35.3) | 1.68 (1.49-1.87) |  |
| Belarus | 4322 (2910-5820) | 33.34 (22.44-44.9) | 5182 (3182-7541) | 33.21 (20.68-48.02) | -0.68 (-0.97--0.38) |  |
| Belgium | 8079 (6041-10136) | 54.84 (41.54-68.8) | 6126 (4550-7769) | 28.58 (21.47-36.08) | -1.87 (-2.1--1.64) |  |
| Belize | 6 (4-7) | 5.66 (4.18-7.19) | 30 (22-39) | 8.75 (6.34-11.29) | 1.82 (1.33-2.31) |  |
| Benin | 43 (19-71) | 1.99 (0.87-3.27) | 189 (103-298) | 3.13 (1.69-4.9) | 0 (0-0) |  |
| Bermuda | 29 (22-36) | 44.85 (33.93-56.42) | 33 (23-43) | 27 (19.37-35.9) | 0 (0-0) |  |
| Bhutan | 11 (5-18) | 3.43 (1.51-5.57) | 4 (1-9) | 0.56 (0.15-1.34) | 0 (0-0) |  |
| Bosnia and Herzegovina | 581 (336-829) | 12.79 (7.26-18.28) | 1714 (1170-2417) | 28.5 (19.44-40.24) | 3 (2.7-3.29) |  |
| Botswana | 53 (30-79) | 7.89 (4.52-11.72) | 143 (84-224) | 7.85 (4.78-12.22) | 0 (0-0) |  |
| Brunei Darussalam | 9 (6-13) | 6.81 (4.6-9.59) | 6 (1-12) | 1.38 (0.12-2.65) | -1.6 (-3.51-0.34) |  |
| Bulgaria | 5476 (3967-6990) | 44.48 (32.28-56.73) | 8166 (6168-10710) | 62.87 (47.48-82.16) | 1.49 (1.34-1.63) |  |
| Burkina Faso | 247 (159-348) | 5.42 (3.48-7.57) | 697 (463-956) | 7.06 (4.72-9.69) | 0 (0-0) |  |
| Burundi | 386 (225-563) | 14.91 (8.93-21.55) | 536 (298-872) | 9.02 (5.17-14.54) | 0 (0-0) |  |
| Cabo Verde | 7 (4-11) | 3.18 (1.84-4.75) | 45 (28-65) | 9.7 (6.11-13.93) | 0 (0-0) |  |
| Cambodia | 189 (84-330) | 3.39 (1.49-5.93) | 2645 (1685-3795) | 18.36 (11.79-26.23) | 5.15 (4.79-5.5) |  |
| Cameroon | 339 (197-507) | 6.86 (3.99-10.3) | 1641 (1005-2410) | 11.25 (6.95-16.47) | 0 (0-0) |  |
| Canada | 9358 (6486-12582) | 29.54 (20.69-39.61) | 15737 (11686-19965) | 24.82 (18.69-31.34) | -0.17 (-0.33--0.01) |  |
| Central African Republic | 120 (52-188) | 8.76 (3.96-13.75) | 145 (58-266) | 5.01 (2.06-9.09) | 0 (0-0) |  |
| Chad | 29 (4-71) | 0.96 (0.14-2.32) | 301 (78-578) | 4.44 (1.12-8.48) | 0 (0-0) |  |
| Chile | 2229 (1727-2773) | 21.22 (16.35-26.52) | 5927 (4492-7531) | 23.66 (17.99-29.99) | 0.83 (0.56-1.1) |  |
| China | 181730 (127133-236829) | 18.89 (13.29-24.64) | 421731 (299868-578280) | 20.34 (14.46-27.96) | 0.35 (0.05-0.66) |  |
| Colombia | 1823 (1397-2294) | 8.3 (6.34-10.5) | 4419 (3172-5942) | 8.04 (5.77-10.81) | -0.35 (-0.65--0.06) |  |
| Congo | 107 (43-182) | 8.77 (3.45-15.05) | 454 (252-701) | 13.98 (7.89-21.24) | 0 (0-0) |  |
| Cook Islands | 0 (0-1) | 1.22 (0-3.55) | 2 (1-3) | 8.79 (6.04-12.14) | 0 (0-0) |  |
| Costa Rica | 191 (149-250) | 10.08 (7.77-13.15) | 896 (674-1176) | 16.32 (12.26-21.42) | 1.52 (1.12-1.93) |  |
| Croatia | 2692 (1824-3557) | 43.83 (29.39-57.98) | 3763 (2771-4930) | 44.71 (33.65-58.79) | -0.02 (-0.3-0.25) |  |
| Cuba | 1154 (851-1500) | 11.1 (8.19-14.42) | 2645 (1953-3561) | 14.48 (10.64-19.38) | 1.2 (1.02-1.38) |  |
| Cyprus | 210 (155-278) | 27.11 (19.61-36.09) | 381 (273-503) | 18.73 (13.49-24.56) | -0.7 (-1.02--0.38) |  |
| Czech | 11396 (8560-14376) | 83.96 (63.88-105.64) | 10177 (7543-13231) | 50.58 (37.82-66.53) | -2.05 (-2.26--1.85) |  |
| C么te d'Ivoire | 196 (101-315) | 4.19 (2.17-6.67) | 723 (432-1164) | 5.59 (3.35-8.71) | 0 (0-0) |  |
| Democratic Korea | 2503 (1574-3953) | 13.09 (8.26-20.23) | 4014 (2499-6661) | 11.69 (7.27-19.31) | -0.15 (-0.31-0.02) |  |
| Denmark | 4300 (3182-5465) | 56.76 (42.37-72.06) | 4015 (3017-5206) | 35.08 (26.5-44.96) | -2.05 (-2.39--1.71) |  |
| Djibouti | 5 (1-9) | 2.55 (0.65-5.08) | 5 (1-11) | 0.54 (0.12-1.16) | 0 (0-0) |  |
| Dominica | 8 (6-10) | 13.01 (9.44-17.15) | 14 (10-19) | 16.16 (11.48-22.2) | 0.92 (0.75-1.1) |  |
| Dominican | 283 (202-390) | 6.29 (4.41-8.72) | 986 (695-1377) | 9.32 (6.59-13.08) | 1.52 (1.27-1.77) |  |
| Ecuador | 212 (131-297) | 3.34 (2.11-4.68) | 1156 (758-1667) | 6.71 (4.4-9.7) | 3 (2.23-3.78) |  |
| Egypt | 77 (48-110) | 0.2 (0.12-0.3) | 324 (215-464) | 0.39 (0.26-0.56) | 3.3 (2.88-3.72) |  |
| El Salvador | 127 (94-169) | 3.66 (2.66-4.88) | 425 (298-568) | 6.96 (4.88-9.3) | 1.68 (1.38-1.98) |  |
| Equatorial Guinea | 13 (4-23) | 5.82 (2-10.17) | 96 (49-153) | 15.15 (7.93-23.79) | 0 (0-0) |  |
| Estonia | 555 (363-752) | 27.71 (18.29-37.69) | 746 (461-1059) | 31.51 (20.41-43.53) | 0.29 (0.04-0.54) |  |
| Eswatini | 38 (22-56) | 11.49 (6.88-16.77) | 109 (61-166) | 15.77 (9.07-23.91) | 0 (0-0) |  |
| Ethiopia | 2004 (596-3829) | 8.41 (2.57-15.96) | 6351 (3518-10267) | 12.72 (7.04-20.64) | 0 (0-0) |  |
| Federative Brazil | 9526 (7385-11783) | 9.19 (7.1-11.38) | 36226 (28825-44537) | 14.11 (11.24-17.36) | 1.33 (1.02-1.63) |  |
| Fiji | 17 (9-25) | 3.4 (1.8-5.1) | 42 (23-62) | 4.83 (2.67-7.09) | 1.4 (0.83-1.98) |  |
| Finland | 1732 (1276-2227) | 25.55 (19.07-32.8) | 2176 (1626-2764) | 19.69 (14.88-24.83) | -0.66 (-0.82--0.5) |  |
| French Republic | 45584 (34459-58293) | 57.78 (44.29-73.85) | 42880 (33079-54151) | 33.66 (25.85-42.17) | -1.66 (-1.73--1.58) |  |
| Gabonese Republic | 153 (72-241) | 25.46 (11.97-40.09) | 270 (165-398) | 22.78 (13.89-33.06) | 0 (0-0) |  |
| Gambia | 4 (1-7) | 0.92 (0.35-1.62) | 24 (14-36) | 2.13 (1.26-3.23) | 0 (0-0) |  |
| Georgia | 731 (430-1123) | 11.61 (6.84-17.78) | 1118 (770-1535) | 20.65 (14.57-28.08) | 3.71 (3.04-4.38) |  |
| Germany | 81048 (61366-101898) | 66.67 (51.01-83.58) | 64409 (50071-81598) | 37.31 (29.32-46.95) | -2.09 (-2.18--1.99) |  |
| Ghana | 313 (162-489) | 4.47 (2.34-6.95) | 1186 (714-1765) | 6.31 (3.81-9.46) | 0 (0-0) |  |
| Grand Duchy of Luxembourg | 341 (261-429) | 64.67 (49.83-81.06) | 316 (242-396) | 30.47 (23.51-38.21) | -2.31 (-2.49--2.12) |  |
| Greenland | 24 (14-34) | 53.91 (31.93-79.35) | 31 (19-44) | 40.84 (25.51-58.35) | 0 (0-0) |  |
| Grenada | 9 (6-12) | 13.39 (9.6-17.65) | 22 (17-30) | 18.61 (13.92-24.85) | 0.87 (0.58-1.16) |  |
| Guam | 5 (0-14) | 5.05 (0.07-13.65) | 22 (5-38) | 10.94 (2.43-19.29) | 0 (0-0) |  |
| Guatemala | 127 (96-162) | 2.83 (2.13-3.63) | 545 (383-733) | 4.32 (3.02-5.86) | 1.35 (1-1.71) |  |
| Guinea | 32 (11-58) | 0.91 (0.31-1.67) | 116 (47-211) | 1.87 (0.77-3.35) | 0 (0-0) |  |
| Guinea-Bissau | 30 (16-44) | 6.7 (3.52-9.79) | 63 (38-92) | 6.93 (4.32-9.92) | 0 (0-0) |  |
| Guyana | 73 (53-93) | 16.34 (12.08-21) | 109 (76-151) | 15.16 (10.55-20.89) | 0.08 (-0.19-0.34) |  |
| Haiti | 516 (323-751) | 13.83 (8.58-20.13) | 1096 (677-1710) | 12.8 (7.82-20.15) | 0 (-0.08-0.09) |  |
| Hashemite Jordan | 10 (7-14) | 0.62 (0.39-0.88) | 48 (30-69) | 0.53 (0.33-0.77) | -0.34 (-0.9-0.22) |  |
| Hellenic Republic | 4118 (3075-5266) | 27.79 (21.1-35.38) | 4921 (3657-6182) | 23.31 (17.43-29.08) | -0.82 (-0.94--0.7) |  |
| Honduras | 61 (43-83) | 2.47 (1.74-3.32) | 236 (161-338) | 3.34 (2.32-4.74) | 0.94 (0.84-1.05) |  |
| Hungary | 9401 (7009-12203) | 65.27 (48.92-84.48) | 10246 (7519-13204) | 57.6 (42.55-73.9) | -0.59 (-0.79--0.4) |  |
| Iceland | 43 (29-60) | 15.92 (10.76-21.9) | 107 (78-139) | 19.5 (14.28-25.21) | 0.9 (0.64-1.17) |  |
| India | 10343 (5298-15314) | 1.83 (0.93-2.73) | 40968 (27944-56671) | 3.12 (2.12-4.33) | 0 (0-0) |  |
| Indonesia | 906 (304-1543) | 0.71 (0.23-1.22) | 2471 (771-4582) | 0.86 (0.27-1.59) | 0.15 (-0.1-0.4) |  |
| Iran | 0 (0-0) | 0 (0-0) | 851 (585-1139) | 0.94 (0.64-1.27) | 37.4 (30.56-44.59) |  |
| Iraq | 33 (23-46) | 0.35 (0.24-0.49) | 76 (48-113) | 0.26 (0.16-0.37) | -1.01 (-1.24--0.77) |  |
| Ireland | 1944 (1483-2441) | 49.63 (38.47-62.13) | 2062 (1545-2646) | 27.36 (20.63-34.95) | -2.09 (-2.26--1.92) |  |
| Italy | 42096 (32248-51795) | 49.27 (38.06-60.55) | 38064 (29398-47424) | 28.84 (22.6-35.54) | -1.89 (-2.04--1.74) |  |
| Jamaica | 109 (77-150) | 6.24 (4.4-8.61) | 366 (236-535) | 11.87 (7.68-17.38) | 2.27 (1.73-2.81) |  |
| Japan | 61413 (46839-76577) | 36.28 (27.77-45.24) | 72774 (53116-93003) | 25.8 (19.14-32.94) | -1.38 (-1.48--1.28) |  |
| Kazakhstan | 3682 (2492-4977) | 26.17 (17.52-35.44) | 2758 (1950-3700) | 13.98 (9.88-18.93) | -2.02 (-2.17--1.88) |  |
| Kenya | 487 (273-713) | 5.2 (3.04-7.58) | 1945 (1230-2847) | 7.22 (4.5-10.37) | 0 (0-0) |  |
| Kiribati | 1 (0-3) | 2.71 (0.51-5.57) | 1 (0-3) | 1.36 (0.16-3.59) | -2.93 (-3.33--2.52) |  |
| Korea | 9084 (6723-12044) | 25.68 (19-33.76) | 18310 (13587-24103) | 20.24 (14.99-26.71) | -0.96 (-1.17--0.76) |  |
| Kyrgyz Republic | 354 (255-466) | 10.73 (7.73-14.24) | 399 (285-555) | 6.77 (4.81-9.53) | -1.25 (-1.51--1) |  |
| Lao People's Democratic Republic | 312 (138-536) | 12.45 (5.38-21.59) | 1147 (712-1716) | 20.32 (12.74-30.54) | 1.75 (1.4-2.11) |  |
| Latvia | 959 (642-1275) | 27.49 (18.76-36.49) | 1246 (812-1742) | 36.52 (24.64-49.55) | 0.8 (0.61-0.99) |  |
| Lebanese Republic | 150 (86-213) | 6.37 (3.7-9.04) | 197 (130-284) | 3.34 (2.2-4.83) | -1.53 (-1.77--1.3) |  |
| Lesotho | 48 (26-77) | 5.18 (2.83-8.36) | 173 (94-269) | 13.59 (7.37-21.17) | 0 (0-0) |  |
| Liberia | 69 (45-97) | 5.6 (3.63-7.86) | 130 (66-226) | 4.88 (2.49-8.4) | 0 (0-0) |  |
| Lithuania | 1210 (795-1645) | 27.22 (18.01-36.99) | 1659 (1070-2316) | 33.11 (22.26-44.68) | 0.84 (0.43-1.26) |  |
| Madagascar | 253 (104-405) | 4.34 (1.85-7) | 443 (198-748) | 3.02 (1.36-5.01) | 0 (0-0) |  |
| Malawi | 77 (38-119) | 1.75 (0.88-2.72) | 282 (177-451) | 3.1 (1.95-4.88) | 0 (0-0) |  |
| Malaysia | 718 (460-1008) | 6.81 (4.31-9.61) | 1325 (776-1966) | 4.28 (2.5-6.4) | -2.12 (-2.58--1.65) |  |
| Maldives | 1 (0-2) | 0.44 (0-1.41) | 3 (1-6) | 0.6 (0.17-1.25) | -2.44 (-4.3--0.54) |  |
| Mali | 86 (54-126) | 2.08 (1.27-3.11) | 203 (132-297) | 2.17 (1.41-3.21) | 0 (0-0) |  |
| Malta | 99 (69-132) | 23.13 (16.14-31.01) | 170 (124-216) | 19.29 (14.04-24.66) | -0.42 (-0.61--0.23) |  |
| Marshall Islands | 1 (0-1) | 3.86 (1.77-6.38) | 2 (1-4) | 5.29 (2.59-8.33) | 1.06 (0.96-1.17) |  |
| Mauritania | 0 (0-0) | 0 (0-0) | 0 (0-0) | 0 (0-0) | 0 (0-0) |  |
| Mauritius | 58 (39-77) | 6.98 (4.75-9.38) | 245 (168-328) | 13.33 (9.14-18.02) | 0 (0-0) |  |
| Micronesia | 5 (3-8) | 9.1 (5.03-13.84) | 5 (2-8) | 5.12 (2.69-8.53) | -2.24 (-2.39--2.09) |  |
| Moldova | 2028 (1399-2691) | 43.84 (30.14-58.28) | 2474 (1704-3374) | 42.16 (29.35-57.47) | 0 (-0.42-0.43) |  |
| Mongolia | 64 (37-98) | 5.29 (2.98-8.15) | 356 (239-507) | 12.23 (8.14-17.4) | 3.9 (3.53-4.28) |  |
| Montenegro | 167 (113-233) | 25.82 (17.51-36.18) | 286 (208-391) | 29.34 (21.35-40.01) | 0.56 (0.45-0.67) |  |
| Morocco | 56 (37-87) | 0.3 (0.19-0.46) | 111 (67-174) | 0.28 (0.17-0.44) | 0 (0-0) |  |
| Mozambique | 19 (1-45) | 0.28 (0.02-0.67) | 197 (96-344) | 1.52 (0.75-2.67) | 0 (0-0) |  |
| Namibia | 41 (19-65) | 5.52 (2.44-8.82) | 179 (106-260) | 11.15 (6.8-16.04) | 0 (0-0) |  |
| Nauru | 1 (0-2) | 15.41 (5.77-29.1) | 1 (1-2) | 16.62 (8.81-25.22) | 0 (0-0) |  |
| Nepal | 19 (1-52) | 0.15 (0-0.43) | 631 (266-1055) | 2.44 (1.03-4.12) | 0 (0-0) |  |
| Netherlands | 9827 (7460-12374) | 50.79 (38.82-63.91) | 13745 (10654-17446) | 41.28 (31.99-52.64) | -0.65 (-0.86--0.44) |  |
| New Zealand | 2760 (1885-3662) | 72.91 (50.8-96.58) | 3047 (2283-3947) | 38.38 (28.88-49.76) | -2.13 (-2.25--2.01) |  |
| Nicaragua | 61 (43-84) | 3.11 (2.25-4.27) | 298 (212-413) | 5.31 (3.76-7.27) | 2.32 (1.97-2.68) |  |
| Niger | 4 (0-10) | 0.1 (0.01-0.28) | 28 (6-70) | 0.28 (0.06-0.71) | 0 (0-0) |  |
| Nigeria | 2404 (1476-3455) | 5.11 (3.13-7.3) | 6488 (4201-9447) | 6.52 (4.31-9.2) | 0 (0-0) |  |
| Niue | 0 (0-0) | 7.9 (1.23-14.31) | 0 (0-0) | 11.37 (4.56-17.15) | 0 (0-0) |  |
| North Macedonia | 602 (430-805) | 31.04 (22.13-41.48) | 977 (696-1351) | 28.64 (20.6-39.32) | -0.44 (-0.59--0.3) |  |
| Northern Mariana Islands | 2 (0-5) | 6.88 (0.11-15.85) | 6 (1-11) | 9.98 (1.33-18.84) | 0 (0-0) |  |
| Norway | 1743 (1187-2389) | 28.88 (20.08-39.54) | 2420 (1791-3169) | 25.74 (19.08-33.5) | -0.27 (-0.54-0) |  |
| Pakistan | 227 (51-439) | 0.35 (0.07-0.68) | 1313 (616-2176) | 0.83 (0.39-1.39) | 0 (0-0) |  |
| Palau | 0 (0-1) | 3.37 (0.43-7.88) | 1 (0-2) | 4.31 (0.93-9.06) | 0 (0-0) |  |
| Palestine | 28 (18-42) | 2.84 (1.78-4.27) | 72 (49-101) | 2.23 (1.49-3.11) | 0 (0-0) |  |
| Panama | 149 (114-185) | 9.13 (6.96-11.4) | 583 (398-767) | 13.21 (9.03-17.39) | 1.56 (1.34-1.78) |  |
| Papua New Guinea | 27 (16-41) | 1.03 (0.62-1.57) | 56 (32-83) | 0.76 (0.42-1.13) | -0.66 (-1.25--0.07) |  |
| Paraguay | 258 (193-342) | 10.67 (7.86-14.06) | 1025 (702-1407) | 16.56 (11.32-22.69) | 1.34 (1.2-1.48) |  |
| People's Democratic Algeria | 48 (31-67) | 0.34 (0.22-0.48) | 196 (133-279) | 0.5 (0.33-0.71) | 1.52 (1.4-1.65) |  |
| Peru | 1258 (772-1790) | 9.31 (5.77-13.34) | 3949 (2513-5730) | 11.33 (7.2-16.34) | 0.93 (0.58-1.29) |  |
| Philippines | 6202 (4409-7934) | 15.95 (11.31-20.6) | 22761 (16844-29697) | 23.58 (17.51-30.82) | 1.43 (1.36-1.51) |  |
| Plurinational State of Bolivia | 392 (219-592) | 10.77 (6.09-16.14) | 1172 (780-1747) | 11.87 (7.95-17.75) | 0.61 (0.37-0.85) |  |
| Poland | 15477 (11717-19265) | 35.37 (26.73-44.03) | 31456 (23867-39180) | 46.55 (35.71-58.03) | 1.02 (0.85-1.19) |  |
| Portuguese Republic | 6669 (5034-8538) | 49.58 (37.62-63.01) | 8362 (6481-10690) | 38.74 (30.01-49.5) | -0.78 (-1--0.55) |  |
| Principality of Andorra | 38 (24-58) | 64.12 (40.52-98.61) | 55 (35-78) | 36.55 (23.15-51.47) | -1.57 (-1.78--1.37) |  |
| Principality of Monaco | 19 (0-40) | 31.64 (0.7-64.55) | 31 (1-63) | 36.35 (1.57-73.52) | 0 (0-0) |  |
| Puerto Rico | 551 (410-723) | 15.33 (11.43-20.14) | 875 (640-1139) | 16.22 (11.91-21.17) | 0 (0-0) |  |
| Romania | 7789 (5757-10283) | 27.44 (20.36-36.27) | 16502 (12108-21823) | 48.7 (36.2-64.77) | 1.71 (1.49-1.93) |  |
| Russian Federation | 55340 (40615-69587) | 30.07 (22.05-37.78) | 65497 (45831-85685) | 28.62 (20.64-37.23) | -0.5 (-0.88--0.11) |  |
| Rwanda | 607 (342-874) | 18.14 (10.87-26.07) | 888 (509-1335) | 11.71 (6.85-17.52) | 0 (0-0) |  |
| Saint Kitts and Nevis | 4 (0-6) | 12.75 (0.17-17.87) | 7 (0-17) | 9.21 (0.08-22.78) | 0 (0-0) |  |
| Saint Lucia | 15 (12-19) | 16.94 (13.06-20.8) | 37 (27-48) | 15.5 (11.29-20.25) | -0.35 (-0.49--0.22) |  |
| Saint Vincent and the Grenadines | 7 (5-10) | 9.47 (6.37-13.01) | 28 (21-36) | 19.74 (14.78-25.21) | 2.4 (2.17-2.64) |  |
| Samoa | 4 (2-5) | 3.91 (2.28-5.5) | 4 (2-7) | 2.69 (1.4-4.14) | -1.45 (-1.82--1.07) |  |
| San Marino | 14 (0-23) | 41.42 (0.1-68.83) | 15 (0-27) | 21.46 (0.09-39.35) | 0 (0-0) |  |
| Sao Tome and Principe | 6 (4-9) | 9.34 (5.82-13.24) | 18 (12-25) | 15.44 (10.47-21.15) | 0 (0-0) |  |
| Saudi Arabia | 28 (8-50) | 0.33 (0.08-0.59) | 89 (14-181) | 0.26 (0.04-0.52) | 0 (0-0) |  |
| Senegal | 34 (8-68) | 0.95 (0.22-1.93) | 58 (17-122) | 0.66 (0.18-1.37) | 0 (0-0) |  |
| Serbia | 4263 (2738-6187) | 36.65 (22.99-52.86) | 5828 (4084-7878) | 37.17 (26.03-49.89) | 0.07 (-0.07-0.2) |  |
| Seychelles | 7 (5-10) | 12.91 (8.93-17.28) | 21 (15-29) | 16.76 (11.75-22.47) | 0 (0-0) |  |
| Sierra Leone | 94 (62-140) | 4.4 (2.92-6.51) | 165 (104-234) | 3.9 (2.44-5.48) | 0 (0-0) |  |
| Singapore | 235 (153-329) | 8.98 (5.85-12.49) | 412 (271-580) | 4.74 (3.12-6.64) | -2.75 (-3.2--2.31) |  |
| Slovak Republic | 4139 (3104-5404) | 69.4 (52.14-90.06) | 5170 (3831-6707) | 55.43 (40.89-71.55) | -0.8 (-0.88--0.72) |  |
| Slovenia | 1065 (737-1418) | 43.32 (30.08-57.87) | 832 (368-1367) | 20.05 (9.17-32.89) | -2.84 (-3--2.69) |  |
| Socialist Viet Nam | 246 (48-512) | 0.55 (0.11-1.16) | 25416 (17080-34183) | 23.53 (15.99-31.32) | 13.11 (11.69-14.55) |  |
| Solomon Islands | 2 (0-3) | 0.79 (0.19-1.68) | 8 (4-15) | 1.72 (0.72-3.01) | 3.7 (2.93-4.47) |  |
| Somalia | 0 (0-0) | 0 (0-0) | 0 (0-0) | 0 (0-0) | 0 (0-0) |  |
| South Africa | 3403 (2383-4535) | 14.31 (10.17-19.42) | 8168 (5947-10839) | 15.91 (11.6-20.92) | 0 (0-0) |  |
| South Sudan | 17 (1-45) | 0.57 (0.02-1.55) | 24 (4-62) | 0.47 (0.08-1.19) | 0 (0-0) |  |
| Spain | 21828 (16817-27594) | 42.49 (33-53.53) | 28215 (21487-35956) | 32.17 (24.81-40.85) | -1.04 (-1.19--0.89) |  |
| Sri Lanka | 294 (205-387) | 2.35 (1.65-3.13) | 864 (499-1315) | 3.15 (1.83-4.78) | 1.06 (0.73-1.39) |  |
| State of Eritrea | 52 (13-96) | 3.24 (0.85-5.97) | 108 (37-206) | 2.79 (0.93-5.24) | 0 (0-0) |  |
| State of Israel | 280 (133-475) | 5.97 (2.84-10.21) | 889 (565-1287) | 7.74 (4.94-11.23) | 0.98 (0.62-1.34) |  |
| State of Kuwait | 0 (0-0) | 0 (0-0) | 5 (0-13) | 0.11 (0-0.3) | 0 (0-0) |  |
| State of Libya | 0 (0-1) | 0.01 (0-0.02) | 61 (36-98) | 0.93 (0.52-1.5) | 11.5 (7.61-15.53) |  |
| State of Qatar | 2 (2-4) | 1.07 (0.65-1.64) | 17 (11-25) | 0.79 (0.47-1.18) | 0 (0-0) |  |
| Sudan | 242 (145-393) | 2.24 (1.35-3.62) | 0 (0-1) | 0 (0-0) | 0 (0-0) |  |
| Sultanate of Oman | 2 (1-4) | 0.21 (0.1-0.38) | 7 (4-12) | 0.23 (0.12-0.37) | 0 (0-0) |  |
| Suriname | 39 (28-50) | 13.56 (9.77-17.75) | 89 (60-127) | 13.5 (9.08-19.31) | 0.31 (0.09-0.54) |  |
| Sweden | 4545 (3223-5881) | 33.66 (24.94-43.38) | 4593 (3228-6030) | 23.46 (16.71-30.44) | -0.86 (-1.01--0.71) |  |
| Swiss Confederation | 3621 (2755-4563) | 36.89 (28.55-46.3) | 3491 (2638-4494) | 20.45 (15.63-26.18) | -1.96 (-2.17--1.75) |  |
| Syrian Arab Republic | 89 (58-128) | 1.37 (0.87-1.99) | 96 (44-165) | 0.65 (0.3-1.11) | 0 (0-0) |  |
| Taiwan (Province of China) | 4592 (3617-5657) | 25.45 (20.06-31.37) | 8557 (6604-10852) | 21.5 (16.58-27.3) | -1.33 (-1.66--0.99) |  |
| Tajikistan | 162 (116-224) | 4.82 (3.44-6.66) | 192 (122-340) | 2.23 (1.41-3.77) | -2.83 (-3.41--2.23) |  |
| Thailand | 5661 (4050-7569) | 13.39 (9.58-17.77) | 20281 (13078-28455) | 20.03 (13.06-28.05) | 0.99 (0.83-1.14) |  |
| the Congo | 781 (280-1349) | 4.38 (1.59-7.55) | 1752 (674-3377) | 3.94 (1.53-7.43) | 0 (0-0) |  |
| Timor-Leste | 10 (5-18) | 2.69 (1.32-4.54) | 55 (34-80) | 5.98 (3.66-8.67) | 2.37 (2.05-2.69) |  |
| Togolese Republic | 44 (25-68) | 3.07 (1.79-4.78) | 164 (89-271) | 3.56 (1.95-5.8) | 0 (0-0) |  |
| Tokelau | 0 (0-0) | 3.89 (1.41-6.81) | 0 (0-0) | 5.96 (2.72-10.2) | 0 (0-0) |  |
| Tonga | 1 (0-1) | 1.11 (0.46-1.79) | 1 (0-2) | 0.97 (0.31-1.86) | -0.86 (-1.72-0.02) |  |
| Trinidad and Tobago | 131 (98-166) | 14.66 (10.85-18.61) | 341 (234-488) | 18 (12.35-25.59) | 1.18 (0.95-1.4) |  |
| Tunisia | 75 (50-105) | 1.33 (0.88-1.87) | 259 (165-387) | 1.86 (1.19-2.75) | 0 (0-0) |  |
| Turkey | 2371 (1639-3300) | 5.69 (3.88-7.9) | 4415 (2972-6156) | 4.52 (3.06-6.28) | 0 (0-0) |  |
| Turkmenistan | 100 (54-158) | 4.29 (2.28-6.84) | 316 (202-454) | 6.67 (4.18-9.62) | 1.71 (1.38-2.04) |  |
| Tuvalu | 0 (0-0) | 3.02 (1.26-5.6) | 0 (0-1) | 4.11 (2.06-7.09) | 0 (0-0) |  |
| Uganda | 771 (406-1131) | 10.48 (5.71-15.4) | 2683 (1711-3937) | 14.6 (9.35-21.23) | 0 (0-0) |  |
| Ukraine | 25035 (16918-32119) | 35.75 (24.74-45.81) | 19359 (10751-29439) | 26.82 (15.22-40.3) | -0.84 (-1.22--0.47) |  |
| Union of Myanmar | 266 (91-541) | 0.94 (0.33-1.93) | 4568 (2975-6681) | 8.39 (5.48-12.28) | 8.61 (8.1-9.11) |  |
| Union of the Comoros | 1 (0-2) | 0.37 (0.04-0.94) | 6 (2-11) | 1.06 (0.35-1.98) | 0 (0-0) |  |
| United Arab Emirates | 63 (33-103) | 7.38 (3.81-12.44) | 207 (117-361) | 2.72 (1.47-4.75) | 0 (0-0) |  |
| United Great Britain and Northern Ireland | 37122 (27348-46811) | 44.6 (33.5-55.75) | 35607 (27390-43679) | 30.35 (23.5-37.29) | -1.39 (-1.5--1.29) |  |
| United Mexican States | 3117 (2462-3895) | 6.07 (4.72-7.65) | 15784 (11876-20284) | 11.69 (8.8-14.98) | 2.06 (1.79-2.33) |  |
| United States of America | 82897 (58923-110518) | 27.73 (20.12-36.66) | 120535 (94337-148179) | 23.6 (18.59-28.87) | -0.39 (-0.48--0.3) |  |
| United States Virgin Islands | 17 (0-38) | 18.35 (0.08-40.42) | 30 (1-55) | 20.16 (0.99-37.67) | 0 (0-0) |  |
| United Tanzania | 1068 (597-1616) | 8.9 (5.07-13.18) | 3061 (2014-4447) | 10.69 (7.13-15.29) | 0 (0-0) |  |
| Uruguay | 1577 (1234-2003) | 41.82 (32.76-53.33) | 2046 (1537-2594) | 40.46 (30.26-51.44) | -0.1 (-0.21-0.01) |  |
| Uzbekistan | 645 (401-927) | 4.77 (2.93-6.92) | 1595 (1113-2136) | 4.89 (3.36-6.62) | -0.09 (-0.65-0.48) |  |
| Vanuatu | 3 (2-5) | 3.69 (1.94-5.75) | 10 (6-15) | 4.57 (2.65-6.73) | 0.99 (-0.21-2.21) |  |
| Venezuela | 1343 (1055-1671) | 11.68 (9.12-14.48) | 2963 (1865-4264) | 9.67 (6.06-13.85) | -0.47 (-0.75--0.2) |  |
| Yemen | 79 (45-133) | 1.29 (0.72-2.15) | 74 (41-126) | 0.38 (0.21-0.65) | 0 (0-0) |  |
| Zambia | 321 (161-474) | 9.63 (5.16-14.27) | 1507 (745-3554) | 16.74 (8.67-37.18) | 0 (0-0) |  |
| Zimbabwe | 445 (252-633) | 9.77 (5.53-13.82) | 1026 (599-1569) | 11.9 (7-17.81) | 0 (0-0) |  |
